# Obstructive sleep apnea syndrome in polycystic ovary syndrome: a systematic review and meta-analysis

**DOI:** 10.3389/fendo.2025.1532519

**Published:** 2025-04-04

**Authors:** Nur K. Abdul Jafar, Afra Al Balushi, Anuradhaa Subramanian, Siang Ing Lee, Christie J. Bennett, Lisa J. Moran, Aya Mousa, Chau Thien Tay, Helena J. Teede, Darren R. Mansfield

**Affiliations:** ^1^ Monash Centre for Health Research and Implementation, Monash University, Clayton, VIC, Australia; ^2^ Monash Lung and Sleep, Monash Health, Clayton, VIC, Australia; ^3^ Department of Applied Health Sciences, University of Birmingham, Birmingham, United Kingdom; ^4^ Be Active Sleep and Eat (BASE) Facility, Department of Nutrition, Dietetics and Food, Monash University, Notting Hill, VIC, Australia

**Keywords:** polycystic ovary syndrome, obstructive sleep apnea syndrome, OSAS, systematic review, meta-analysis

## Abstract

**Background:**

Polycystic ovary syndrome (PCOS) has been associated with a high prevalence of obstructive sleep apnea syndrome (OSAS). However, the impact of OSAS on the PCOS symptom profile remains unclear. This systematic review and meta-analysis, which informed the 2023 International Evidence-based PCOS Guideline, aims to assess the prevalence and related symptoms of OSAS among females with and without PCOS.

**Methods:**

A systematic search using databases (MEDLINE, Embase, EBM Reviews, PsycInfo and CINAHL) was performed until 16^th^ May 2024. Random-effects restricted maximum likelihood meta-analyses compared OSAS and related symptoms between PCOS and non-PCOS groups. OSAS outcomes were categorized as apnea-hypopnea index (AHI)≥5 only, AHI≥5 with symptoms, AHI≥10 with symptoms and composite OSA (i.e., all AHI cut-offs with and/or without symptoms). Subgroup analyses by body mass index (BMI), age, PCOS diagnostic criteria and ethnicity were performed. Risk of bias and certainty of evidence by the Grading of Recommendations, Assessment, Development and Evaluation (GRADE) framework were conducted.

**Results:**

From 4438 records, 3205 titles/abstracts were screened and 40 were eligible for full-text screening. Eight cross-sectional studies met inclusion criteria and meta-analysis. The pooled prevalence of OSA was 37.0% in PCOS (29.0% adolescents; 40.0% adults) and 6.0% in non-PCOS. Compared with non-PCOS, those with PCOS showed higher risk for composite OSA (odds ratio (OR): 9.52; 95% CI: 3.90 to 23.26; *I* ^2^ = 54.5%; 8 studies, n=942; *P*<0.001) and more pronounced OSAS risk with increasing symptom severity in PCOS (AHI≥5 OR: 3.90; 95% CI: 1.63 to 9.34; AHI≥5 with symptoms OR: 17.95; 95% CI: 6.17 to 52.22; AHI≥10 with symptoms OR: 30.61; 95% CI: 7.99 to 117.25, all *P ≤* 0.0023). Subgroup results showed significantly higher risk of OSAS overall in overweight/obesity, adults and white ethnicity compared with normal weight, adolescent and Asian subgroups, respectively (all *P*<0.001), but independent of PCOS diagnostic criteria.

**Conclusion:**

The prevalence of OSA was higher in PCOS compared with non-PCOS groups, with the risk of OSAS increasing with worse symptom severity. Adults and those of higher BMI and of white ethnicity were at increased risk of OSAS. Hence, identifying and treating OSAS symptoms in PCOS may be beneficial, but further validation of findings is warranted.

## 1 Introduction

Polycystic ovary syndrome (PCOS) is the most common endocrine disorder, affecting up to 13% of reproductive-aged women (1). It is associated with a range of reproductive, metabolic and psychological sequelae (2, 3). Diagnosis of PCOS in adults is based on the presence of two of three features: (i) oligo/amenorrhea; (ii) clinical/biochemical hyperandrogenism and/or (iii) polycystic ovary morphology (4) or elevated serum anti-Müllerian hormone levels, according to the latest 2023 International Evidence-based PCOS Guideline (1). The key pathophysiological drivers of PCOS include hyperandrogenism and intrinsic insulin resistance that is mechanistically distinct from obesity-associated insulin resistance (5, 6).

Several of these pathophysiological risk factors in women with PCOS have been theoretically linked to obstructive sleep apnea (OSA) (7, 8). OSA is characterized by the repetitive collapse of the upper airway during sleep for at least 10 seconds, and associated with oxygen desaturation and/or arousal from sleep (9). Additionally, obstructive sleep apnea syndrome (OSAS) is defined as OSA (apnea-hypopnea index (AHI) ≥5 events per hour) together with features of sleep disturbances, such as excessive daytime sleepiness and/or cardiometabolic comorbidities (e.g., arterial hypertension) (10–12). OSAS and associated snoring is the most common sleep-disordered breathing abnormality (12). In the general population and in PCOS, OSAS has been associated with multiple comorbidities such as obesity, insulin resistance, gestational diabetes, type 2 diabetes, hypertension, and impaired quality of life (12–14). Furthermore, cardiometabolic risk factors such as chronic inflammation, oxidative stress, and impaired fibrinolysis are elevated in PCOS (1) that can lead to higher risk of OSAS.

Past cross-sectional studies have shown that OSAS is highly prevalent in women with PCOS (15–19) compared with the general population of reproductive-aged women (17-75% vs. 9-28%) (9, 10), with lower rates in adolescents with PCOS (0-57%) (20, 21). Previous systematic reviews have also reported higher risk of OSA in PCOS relative to those without PCOS, but this varies between the odds ratios (ORs) of 2.86 and 8.30 (22–26). The highly variable OSA prevalence rates in past reviews relate to the heterogenous measurements, including polysomnography (PSG), cardiorespiratory polygraph and screening questionnaires (e.g., Berlin Questionnaire) (22–26). One recent review also included studies that did not report OSA measurements (26). Indeed, formal sleep studies using PSG assessments are essential for accurate OSA prevalence estimates (11). The wide risk estimates reported in prior reviews are also attributed to the inconsistent study inclusion and exclusion criteria employed, with some including conference abstracts and grey literature (23, 26) and others limiting searches to only peer-reviewed and database-indexed articles (22, 24–26).

To date, there has been no quantitative synthesis of the available evidence linking OSAS and related symptoms with PCOS status. Further, important confounders which may influence this relationship, including body mass index (BMI), age, PCOS diagnostic criteria, and ethnicity, have not been thoroughly interrogated. Higher BMI in adults can lead to physiological changes that contribute to OSA development through accumulation of fat deposits in the upper respiratory tract, thereby narrowing the airway and decreasing muscle activity in this region (7). Some studies however, reported higher prevalence of OSA irrespective of BMI in PCOS, raising the possibility that body fat distribution rather than total body mass may be more useful in determining OSAS risk in PCOS or that other factors may contribute to this association (8, 27, 28). Racial and ethnic disparities in PCOS phenotypes and in OSA diagnosis have also been reported, and may be related to genetic inheritance or health inequities or both (29).

To address these important gaps, we conducted the first systematic review and meta-analysis to comprehensively synthesize the prevalence and related symptoms of OSAS severity in PCOS compared with non-PCOS populations, with inclusion of peer-reviewed published studies that utilized the PSG tool. The methodology applied for this evidence synthesis was aligned with the 2023 International Evidence-based PCOS Guideline (1), which directly informed current recommendations for assessing OSAS in PCOS.

## 2 Methods

### 2.1 Protocol and registration

The protocol was registered *a priori* on the international prospective register of systematic reviews, PROSPERO (CRD42024508308), and reported in accordance with the Preferred Reporting Items for Systematic Reviews and Meta-Analyses (PRISMA) guidelines (30).

### 2.2 Eligibility criteria and search strategy

Eligibility criteria using the Population-Exposure-Comparator-Outcome-Study design (PECOS) framework (**Supplementary Table 1**) were developed in collaboration with the 2023 International PCOS Guideline expert group (1). Studies included were: 1) females of any age, ethnicity, weight and comorbidities (e.g., infertility); 2) had clinically-confirmed diagnosis of PCOS (Rotterdam criteria, National Institutes of Health (NIH) definition or Androgen Excess and PCOS Society (AES) definition); 3) inclusion of a control group without PCOS and 4) used formal sleep studies (i.e., Level 1 (in-laboratory PSG); Level 2 (ambulatory or home-based PSG); or Level 3 (ambulatory limited channel PSG or polygraph)) (11). Eligible study designs included cohort studies (e.g., case-control, controlled cross-sectional) and randomized controlled trial (RCT) studies. Included studies were not restricted by language and year of publication. Other systematic reviews were included initially for screening references to identify additional eligible studies. Studies with self-reported PCOS diagnosis, with other population groups, without a control (non-PCOS) group or without formal sleep study assessments were excluded. Electronic databases MEDLINE, Embase, Evidence-Based Medicine (EBM) Reviews, PsycInfo (all via Ovid) and CINAHL (via EBSCO) were initially searched up to 2^nd^ August 2022 to inform the 2023 PCOS Guideline recommendations (1), with a search update on 16^th^ May 2024 for publication. The search strategy was developed by PCOS guideline development group experts in conjunction with the guideline evidence team (AM, CTT) and co-authors (AS, SL) (Supplementary **Table 2**).

### 2.3 OSAS related symptoms

The presence of symptoms related to OSAS often referred to daytime sleepiness, measured using the validated Epworth Sleepiness Scale (ESS) questionnaire. Using ESS, participants were asked to rate their probability of falling asleep in eight different situations on a scale of 0 (not likely at all) to 3 (extremely likely) (31). Other symptoms related to OSAS include, but not limited to sleep-related complaints (21), choking, witnessed apneic spell, nocturia and hypertension or other cardiovascular complication (16, 18).

### 2.4 Study selection

Screening was undertaken using Covidence (http://www.covidence.org). Duplicates were automatically removed by Covidence, otherwise manually removed by two reviewers (AS, SL, NAJ or AA). Title, abstract and full text screening were conducted in duplicate by two independent reviewers (AS, SL, NAJ or AA) and any disagreement was resolved by a third reviewer (DM), with discussion to reach consensus, where necessary.

### 2.5 Data extraction

Data extraction from each full-text article was completed by one reviewer (NAJ, AS or SL) with independent cross-checking by two reviewers for each article (AA, AS or SL) to ensure accuracy. Using a standardized data extraction template, the following information was extracted from studies: author, year of publication, country of origin, study design, sample size, population characteristics, setting, age, BMI, method of PCOS diagnosis, prevalence and effect estimates, measurements of OSAS severity, outcomes and confounders (e.g., ethnicity).

### 2.6 Quality assessment and GRADE assessment

The quality of the included studies was independently assessed by two reviewers (AS, SL). As no RCTs were identified, quality appraisal was conducted using templates adapted from the Risk Of Bias In Non-randomized Studies of Interventions (ROBINS-I) tool (32). This included assessment of internal validity based on selection bias, performance bias, attrition bias, confounding and other bias (e.g., statistical analysis). Using these criteria, each included study was allocated an overall risk of bias rating of low, moderate or high. The certainty of evidence was independently assessed by two reviewers (AS, SL) using the Grading of Recommendations, Assessment, Development, and Evaluation (GRADE) framework. This was conducted in accordance with the Cochrane GRADE guidelines and recommendations for outcome-level assessment of risk of bias, inconsistency, indirectness, imprecision and other bias such as publication bias (33).

### 2.7 Statistical analysis

The prevalence estimates of OSA (i.e., all AHI cut-offs with and/or without symptoms) were pooled from the included studies for meta-analysis and presented as forest plots of proportions and 95% confidence intervals (CIs) for PCOS and non-PCOS using random effect models. Meta-analyses were conducted separately for each symptom category of OSAS outcome (Composite OSA, AHI ≥5 only, AHI ≥5 with symptoms and AHI ≥10 with symptoms) and presented as pooled ORs and 95% CIs. Where only a single study reported an outcome, this was included as a single-paper analysis (34). Subgroup analyses were conducted to assess the impact of BMI (normal weight: BMI <25 kg/m^2^ vs. overweight: BMI ≥25 to <30 kg/m^2^ or obesity: BMI ≥30 kg/m^2^) (35), age (adults: 20 to 45 years old vs. adolescents: 13 to 19 years old), PCOS diagnostic criteria (NIH vs. Rotterdam) or ethnicity (White vs. Asian or Mixed) on the relationship between PCOS and OSAS, where applicable. All subgroup differences were determined using Chi-square tests. To further investigate the independent association between PCOS and OSAS in relation to BMI, a sensitive analysis was conducted by pooling together studies that have either adjusted or matched their control (non-PCOS) group for BMI. All meta-analyses and funnel plots were performed in STATA version 18 software (Texas, USA) using random-effects restricted maximum likelihood (REML) models (36). Heterogeneity between studies was assessed by *I* ^2^ statistics where a value >50% was considered substantial heterogeneity (37). Publication bias was assessed by visual inspection of asymmetry of funnel plots to assess small study effects, where applicable (38). If substantial heterogeneity was present, the leave-one-out sensitivity analysis was performed, where each study was sequentially excluded to assess its individual influence on the overall pooled effect estimate (39). Additionally, sensitivity analysis was performed to determine the influence of high risk of bias studies on the overall pooled effect estimate, where applicable. *P*-value <0.05 was considered to be statistically significant.

## 3 Results

### 3.1 Search results and study characteristics

**Figure 1:**
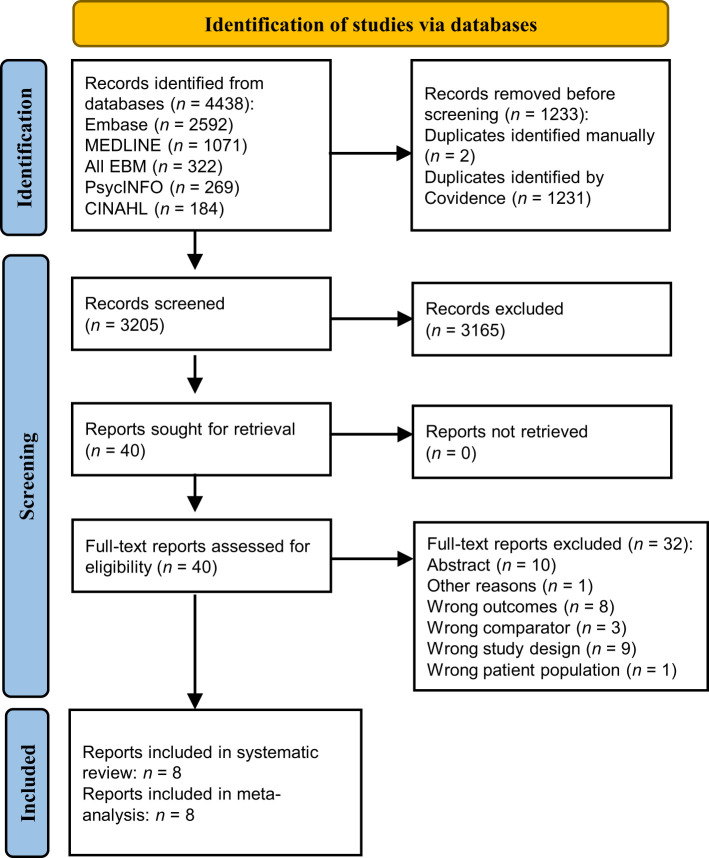
PRISMA flowchart of studies included in the review.

The initial search yielded 4007 records, of which 2487 titles and abstracts were screened and 50 were eligible for full-text screening. From these, eight cross-sectional studies met inclusion criteria and were eligible for meta-analysis. There were no additional studies identified after updating the searches. As shown in **Figure 1**, after updating the searches, the electronic literature search yielded 4438 records. After screening 3205 titles and abstracts and 40 full-texts, eight articles were included in the systematic review. Thirty-two full-text studies were excluded with reasons outlined in **Figure 1** and detailed in **Supplementary Table 3**. Characteristics of included studies are presented in **Table 1**. The included studies were all controlled cross-sectional in design and reported data on the number of cases of OSA in PCOS compared with non-PCOS; thus, all were included in meta-analysis (15–21, 40). A total of 280 females with PCOS (sample sizes range: 18 to 53) and 662 females without PCOS (sample sizes range: 10 to 452) were included. All PCOS diagnoses were confirmed by clinicians, with four studies using the Rotterdam criteria (18, 19, 21, 40) and the other four studies using the NIH criteria (15–17, 20). Six studies were in adult populations (mean age range: 27.9 to 32.3 years) (15–19, 40), while two studies examined adolescent girls (mean age range: 15.0 to 17.1 years) (20, 21). Overall, five studies included populations with overweight and/or obesity (mean BMI range: 26.4 to 44.8 kg/m^2^) (15–17, 20, 21), while one study included normal weight individuals only (40) and two included all weight ranges (18, 19). All eight studies employed PSG to measure OSAS (as this was an inclusion criterion) (15–21, 40), five of which also assessed the presence of other OSAS-related symptoms such as excessive daytime sleepiness (15, 16, 18, 19, 21). The included study populations were mostly of white ethnicity (15–17, 19, 20), with only three studies from Asian (18, 40) or mixed ethnicity (21) populations.

**Table 1:** Study characteristics of included studies (*n* = 8).^1^.

Author, year, country	Population/Setting	Ethnicity	*n* per group	Age (y)	BMI (kg/m^2^)	PCOS criteria	OSA or OSAS related symptoms	Adjusted variables	Risk of bias^2^
Fogel et al. (2001) (15), USA	Advertisement within community (non-PCOS group). Women’s health and Endocrinology hospital clinics (PCOS group)	White	PCOS: *n* = 18 Non-PCOS: *n* = 18	PCOS: 31.1 ± 1.3 Non-PCOS: 32.3 ± 1.3	PCOS: 36.9 ± 1.3 Non-PCOS: 36.9 ± 1.4	NIH	Assessed by PSG OSA: AHI>5, AHI>10, AHI>15 OSAS: AHI>5 with ESS≥10	None adjusted. Age and weight matched groups.	Moderate
Vgontzas et al. (2001) (16), USA	Sleep laboratory (PCOS and non-PCOS groups).	White	PCOS: *n* = 53 Non-PCOS: *n* = 452	PCOS: 30.4± 0.9 Non-PCOS: 32.1± 0.3	PCOS: 38.7 ± 1.1 Non-PCOS: 26.4 ± 0.3	NIH	Assessed by PSG SDB: AHI≥10 with symptoms i.e., daytime sleepiness, hypertension, or other cardiovascular complication	None adjusted.	High
Tasali et al (2008) (17), USA	Advertisement within local community (non-PCOS group). Endocrinology University clinics (PCOS group)	White	PCOS: *n* = 52 Non-PCOS: *n* = 21	PCOS: 29.7 ± 0.7 Non-PCOS: 30.7 ± 1.1	PCOS: 39.2 ± 1.0 Non-PCOS: 36.0 ± 1.5	NIH	Assessed by PSG OSA: AHI≥5	Age, BMI, and ethnicity.	Moderate
Yang et al. (2009) (40), Taiwan	Same community (non-PCOS group). Obstetrics and Gynaecology hospital clinic (PCOS group)	Asian	PCOS: *n* = 18 Non-PCOS: *n* = 10	PCOS: 29.1 ± 1.4 Non-PCOS: 31.6 ± 3.9	PCOS: 21.7 ± 0.6 Non-PCOS: 20.9 ± 0.6	Rotterdam	Assessed by PSG OSA: AHI≥5	None adjusted. Age and weight matched groups.	Moderate
de Sousa et al. (2011) (20), Germany	Outpatient Obesity and Endocrine Children’s Hospital (PCOS and non-PCOS groups)	White	PCOS: *n* = 31 Non- PCOS: *n* = 19	PCOS: 15.0 ± 1.0 Non-PCOS: 15.2 ± 1.1	PCOS: 32.7 ± 6.2 Non-PCOS: 32.4 ± 4.0	1992 NIH	Assessed by PSG OSA: AHI≥5	None adjusted.	Moderate
Nandalike at el. (2012) (21), USA	Sleep disorders centre database (Non-PCOS group). Children’s Hospital (PCOS group)	Mixed	PCOS: *n* = 28 Non-PCOS: *n* = 28	PCOS: 16.8 ± 1.9 Non-PCOS: 17.1 ± 1.8	PCOS: 44.8 ± 8.8 Non-PCOS: 40.2 ± 4.7	Modified 2003 Rotterdam	Assessed by PSG OSA/OSAS: AHI>5 or apnea index >1 (with sleep-related complaints)	None adjusted. Age and BMI Z-score matched groups.	High
Suri et al. (2016) (18), India	Gynaecology Outpatient Department (OPD; Non-PCOS group). OPD and Reproductive Endocrinology College and Hospital Clinic (PCOS group).	Asian	PCOS: *n* = 50 Non-PCOS: *n* = 100	PCOS: 27.9 ± 6.4 Non-PCOS: 28.3 ± 6.1	PCOS: 28.0 ± 4.0 Non-PCOS: 25.3 ± 2.9	Modified 2003 Rotterdam	Assessed by PSG SDB: RDI≥5 with symptoms such as EDS, choking, witnessed apnoeic spell, nocturia, or an RDI >15 with or without associated symptoms.	Adjusted for BMI or waist circumference Age and BMI Z-score matched groups.	High
Hachul et al. (2019) (19), Brazil	Endocrinology Division of the Federal University (PCOS and non-PCOS groups)	White	PCOS: *n* = 30 Non-PCOS: *n* = 14	PCOS: 29.7 ± 1.2 Non-PCOS: 27.9 ± 1.7	PCOS: 34.3 ± 1.1 Non-PCOS: 22.4 ± 1.6	Modified 2003 Rotterdam	Assessed by PSG OSAS: AHI≥5 with sleep complaints or AHI≥15	None adjusted.	Moderate

All studies are of cross-sectional design. AHI, apnea-hypopnea index; BMI, body mass index; EDS, excessive daytime sleepiness; ESS, Epworth sleepiness scale; OSA, obstructive sleep apnea; OSAS, obstructive sleep apnea syndrome; PCOS, polycystic ovary syndrome; PSG, polysomnography; RDI, respiratory disturbance index.

^1^Values are present as mean ± SD and/or range unless otherwise stated.

^2^Overall risk of bias ratings were low, moderate or high.

### 3.2 Outcome measures

As shown in **Table 1**, symptom categories of OSAS outcomes included studies with the following predetermined inclusion criteria: 1) AHI ≥5 only (studies not reporting presence of symptoms related to OSAS) (15, 17, 20, 40), 2) AHI ≥5 with symptoms (15, 18, 19, 21), 3) AHI ≥10 with symptoms (16) and 4) Composite OSA (i.e., all AHI cut-offs with and/or without symptoms) (15–21, 40). Varying degrees of daytime sleepiness definitions were used across four out of eight studies that include: ESS scores ranging from 0–24, with higher scores indicating greater sleepiness (15); ESS score ≥10 considered as excessive daytime sleepiness (18, 19); and a 4-point ESS scale (none, mild, moderate, or severe) (16). Three out of eight studies instead used information on any sleep-related complaints (21) or included other OSAS related symptoms such as choking, witnessed apneic spell, nocturia and hypertension or other cardiovascular complication (16, 18).

### 3.3 Quality assessment and grading

The quality assessment of the included studies revealed that studies were of moderate to high risk of bias (**Table 1**). This was commonly attributed to selection bias, detection bias and overall small sample sizes (as detailed in **Supplementary Table 4**). As shown in **Table 2**, GRADE assessments indicated that evidence certainty for composite OSA was low due to being twice downgraded for the moderate or high risk of bias of all included studies (serious risk of bias) and having no outcome events in two studies (serious imprecision) (20, 40). Moreover, there was moderate evidence certainty for OSAS outcomes; AHI ≥5 only, AHI ≥5 with symptoms and AHI ≥10 with symptoms, again downgraded either once for serious imprecision (20, 40) or for serious risk of bias.

**Table 2:** GRADE assessment and evidence profile.

Comparison: Females with PCOS versus non-PCOS												
	Quality assessment						No. of participants					
No. of studies	Study design	Risk of bias	Inconsistency	Indirectness	Imprecision	Other	PCOS	Non-PCOS	OR, REML random (95% CI)	Favours	Certainty	Importance
Outcome: Composite OSA												
8	Cross-sectional	Serious^2^	Not serious	Not serious	Serious^1^	None	280	662	9.52 (3.90, 23.26)	PCOS	⨁⨁◯◯ Low	CRITICAL
Outcome: AHI≥5 only												
4	Cross-sectional	Not serious	Not serious	Not serious	Serious^1^	None	119	68	3.90 (1.63, 9.34)	PCOS	⨁⨁⨁◯ Moderate	CRITICAL
Outcome: AHI≥5 with symptoms												
4	Cross-sectional	Serious^2^	Not serious	Not serious	Not serious	None	126	160	17.95 (6.17, 52.22)	PCOS	⨁⨁⨁◯ Moderate	CRITICAL
Outcome: AHI≥10 with symptoms												
1	Cross-sectional	Serious^2^	Not serious	Not serious	Not serious	None	53	452	30.61 (7.99, 117.25)	PCOS	⨁⨁⨁◯ Moderate	CRITICAL

AHI, apnea-hypopnea index; CI, confidence interval; GRADE, Grading of Recommendations, Assessment, Development and Evaluations; OSAS, obstructive sleep apnea syndrome; OR, odds ratio; PCOS, polycystic ovary syndrome; REML, restricted maximum likelihood.

^1^Downgraded once due to one or two of the studies had no outcome events.

^2^Downgraded once as the majority of studies being of moderate or high risk of bias.

### 3.4 Meta-analysis

#### 3.4.1 Prevalence and symptom categories of OSAS in PCOS and non-PCOS

**Figure 2:**
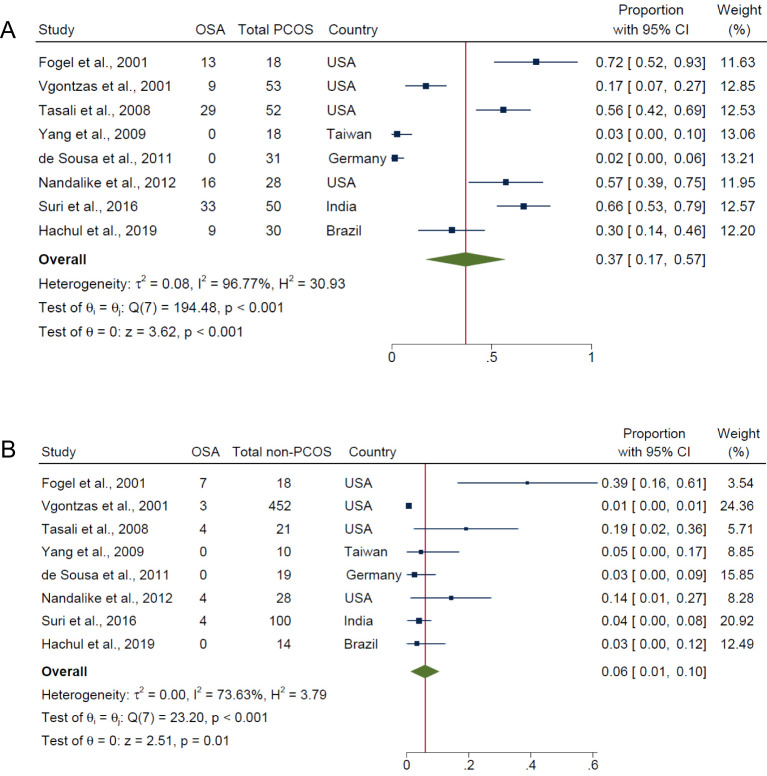
Pooled prevalence of OSA (i.e., all AHI cut-offs with and/or without symptoms) in PCOS and non-PCOS populations. **(A)** PCOS and **(B)** Non-PCOS.

**Figure 2A** demonstrates that the overall pooled prevalence of OSA in PCOS was 37.0% (95% CI: 17.0% to 57.0%; *I* ^2^ = 96.8%; 8 studies, n = 280; *P* < 0.001), compared with a lower pooled prevalence in those without PCOS (6.0%; 95% CI: 1.0% to 10.0%; *I* ^2^ = 73.6%; *P* = 0.01; 8 studies, n = 662; **Figure 2B**). The *I* ^2^ values of 96.8% and 73.6% for PCOS and non-PCOS populations respectively, were both statistically significant for heterogeneity among the studies (both *P* < 0.001). Evidence of publication bias were observed for overall prevalence of OSA in PCOS and non-PCOS populations respectively, based on visual asymmetries of funnel plots (**Supplementary Figure 1**). Sensitivity analysis results showed no difference in significance of the overall findings after exclusion of any individual study from the analysis (**Supplementary Figure 2**).

**Figure 3:**
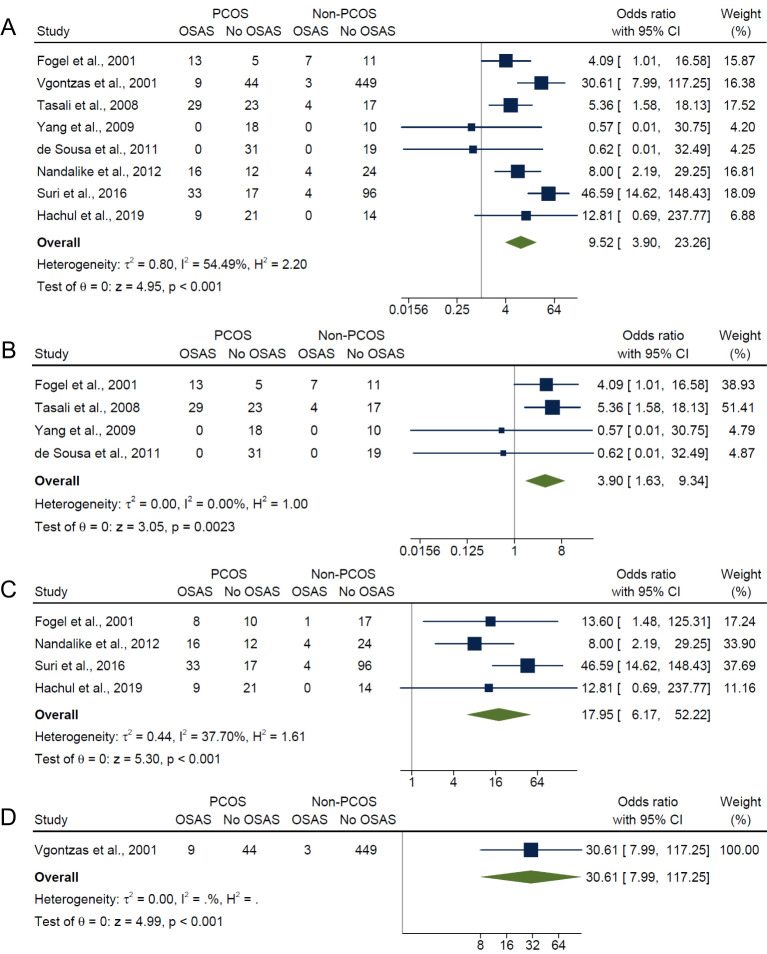
Meta-analyses of symptom categories of OSAS in PCOS compared with non-PCOS. **(A)** Composite OSA, **(B)** AHI≥5 only, **(C)** AHI≥5 with symptoms and **(D)** AHI≥10 with symptoms.

Overall, for composite OSA outcome, individuals with PCOS were approximately 10 times more likely to have OSAS compared with those without PCOS (OR: 9.52; 95% CI: 3.90 to 23.26; *I* ^2^ = 54.5%; *P* < 0.001; 8 studies, n = 942; **Figure 3A**), with only five of the eight studies having adjusted or matched their control group by age, BMI and/or ethnicity (15, 17, 18, 21, 40). Specifically, the risk of OSAS in PCOS compared with non-PCOS groups were more pronounced with presence of symptoms and increasing severity categories of OSAS: AHI ≥5 only (OR: 3.90; 95% CI: 1.63 to 9.34; *I* ^2^ = 0%; *P* = 0.0023; 4 studies, n = 187; **Figure 3B**), AHI ≥5 with symptoms (OR: 17.95; 95% CI: 6.17 to 52.22; *I* ^2^ = 37.7%; *P* < 0.001; 4 studies, n = 286; **Figure 3C**) and AHI ≥10 with symptoms (OR: 30.61; 95% CI: 7.99 to 117.25; *P* < 0.001; 1 study, n = 505; **Figure 3D**). There was no evidence of any publication bias specific for each symptom category of OSAS, as observed from visual symmetries of funnel plots (**Supplementary Figure 3**). Sensitivity analyses results for composite OSA overall showed no difference in significance of the findings after exclusion of any individual study (**Supplementary Figure 4**). Additionally, sensitivity analysis did not change the significance of the results for composite OSA and AHI ≥5 with symptoms after excluding for high risk of bias studies (16, 18, 21) (OR: 4.30; 95% CI: 1.86 to 9.93; *I* ^2^ = 0%; *P* < 0.001; 5 studies, n = 231 and OR: 13.31; 95% CI: 2.27 to 77.95; *I* ^2^ = 0%; *P* < 0.001; 2 studies, n = 80, respectively).

### 3.5 Subgroup analysis

Subgroup analysis were conducted for BMI, age, PCOS diagnostic criteria and ethnicity for composite OSA, AHI ≥5 only and AHI ≥5 with symptoms outcomes respectively. Due to the limited available studies, AHI ≥10 with symptoms outcome was not examined.

#### 3.5.1 Subgroup analysis by BMI

**Figure 4:**
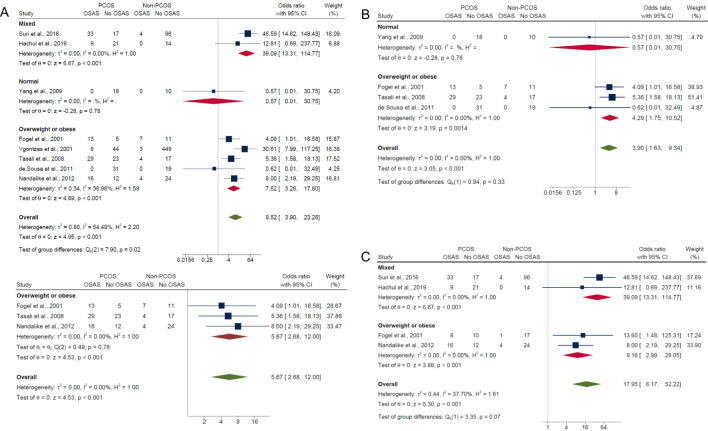
Subgroup analyses of symptom categories of OSAS in PCOS compared with non-PCOS by BMI. **(A)** Composite OSA, **(B)** AHI≥5 only and **(C)** AHI≥5 with symptoms.

In the subgroup analysis by BMI, the overweight/obese category, which included studies having adjusted (17) or matched their control group (15, 21) by age, BMI and/or ethnicity and studies that did not adjust for confounders (16, 20), had significantly higher risk in PCOS compared with non-PCOS groups for composite OSA (OR: 7.62; 95% CI: 3.26 to 17.80; *P* < 0.001; 5 studies, n = 720; **Figure 4A**), AHI ≥5 only (OR: 4.29; 95% CI: 1.75 to 10.52; *P* = 0.0014; 3 studies, n = 159; **Figure 4B**) and AHI ≥5 with symptoms (OR: 9.16; 95% CI: 2.99 to 28.05; *P* < 0.001; 2 studies, n = 92; **Figure 4C**). Sensitivity analysis limited to pooled studies having either adjusted (17) or matched their control group (15, 21) by age, BMI and/or ethnicity also showed significantly higher risk in PCOS compared with non-PCOS groups for composite OSA (OR: 5.67; 95% CI: 2.68 to 12.00; *P* < 0.001; 3 studies, n = 165; **Figure 4A**). In the normal weight subgroup with only one study (40), results were not significant for both composite OSA and AHI ≥5 only (ORs: 0.57; 95% CI: 0.01 to 30.75; *P* = 0.78; n = 28; **Figures 4A, B** respectively). In the mixed weight group (i.e., studies with mixed samples of normal, overweight and obese), there were significantly higher risk of OSAS in PCOS compared with non-PCOS populations for both composite OSA and AHI ≥5 with symptoms (ORs: 39.09; 95% CI: 13.31 to 114.77; *P* < 0.001; 2 studies, n = 194; **Figures 4A, C**).

#### 3.5.2 Subgroup analysis by age

**Figure 5:**
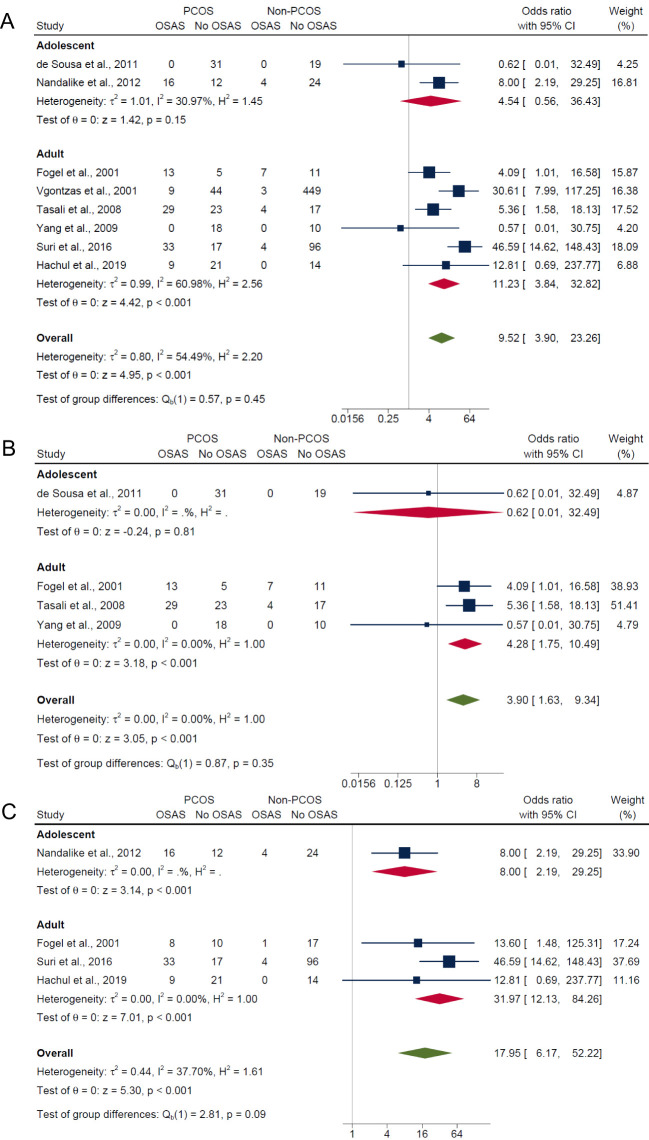
Subgroup analyses of symptom categories of OSAS in PCOS compared with non-PCOS by age. **(A)** Composite OSA, **(B)** AHI≥5 only and **(C)** AHI≥5 with symptoms.

Adolescent PCOS populations had an overall lower pooled prevalence of OSA (29.0%; 95% CI: 0% to 83.0%) compared with adult PCOS populations (40.0%; 95% CI: 17.0% to 63.0%), but differences between groups were not significant (*P* = 0.71) (**Supplementary Figure 5**). For non-PCOS populations, adolescent and adults showed similar pooled prevalence of OSA (both 7.0%) with no significant group differences (*P* = 0.96) (**Supplementary Figure 5**). In adolescents, results showed no significant differences in risk of OSAS between those with PCOS and without PCOS for composite OSA (OR: 4.54; 95% CI: 0.56 to 36.43; *P* = 0.15; 2 studies, n = 106; **Figure 5A**) and AHI ≥5 only (OR: 0.62; 95% CI: 0.01 to 32.49; *P* = 0.81; 1 study, n = 50; **Figure 5B**), but were significant for AHI ≥5 with symptoms where the risk was higher in PCOS (OR: 8.0; 95% CI: 2.19 to 29.25; *P* < 0.001; 1 study, n = 56; **Figure 5C**). In adults, there were significantly higher risk of OSAS in PCOS compared with non-PCOS groups for composite OSA (OR: 11.23; 95% CI: 3.84 to 32.82; *P* < 0.001; 6 studies, n = 836; **Figure 5A**), AHI ≥5 only (OR: 4.28; 95% CI: 1.75 to 10.49; *P* < 0.001; 3 studies, n = 137; **Figure 5B**) and AHI ≥5 with symptoms (OR: 31.97; 95% CI: 12.13 to 84.26; *P* < 0.001; 3 studies, n = 230; **Figure 5C**).

#### 3.5.3 Subgroup analysis by PCOS diagnostic criteria

**Figure 6:**
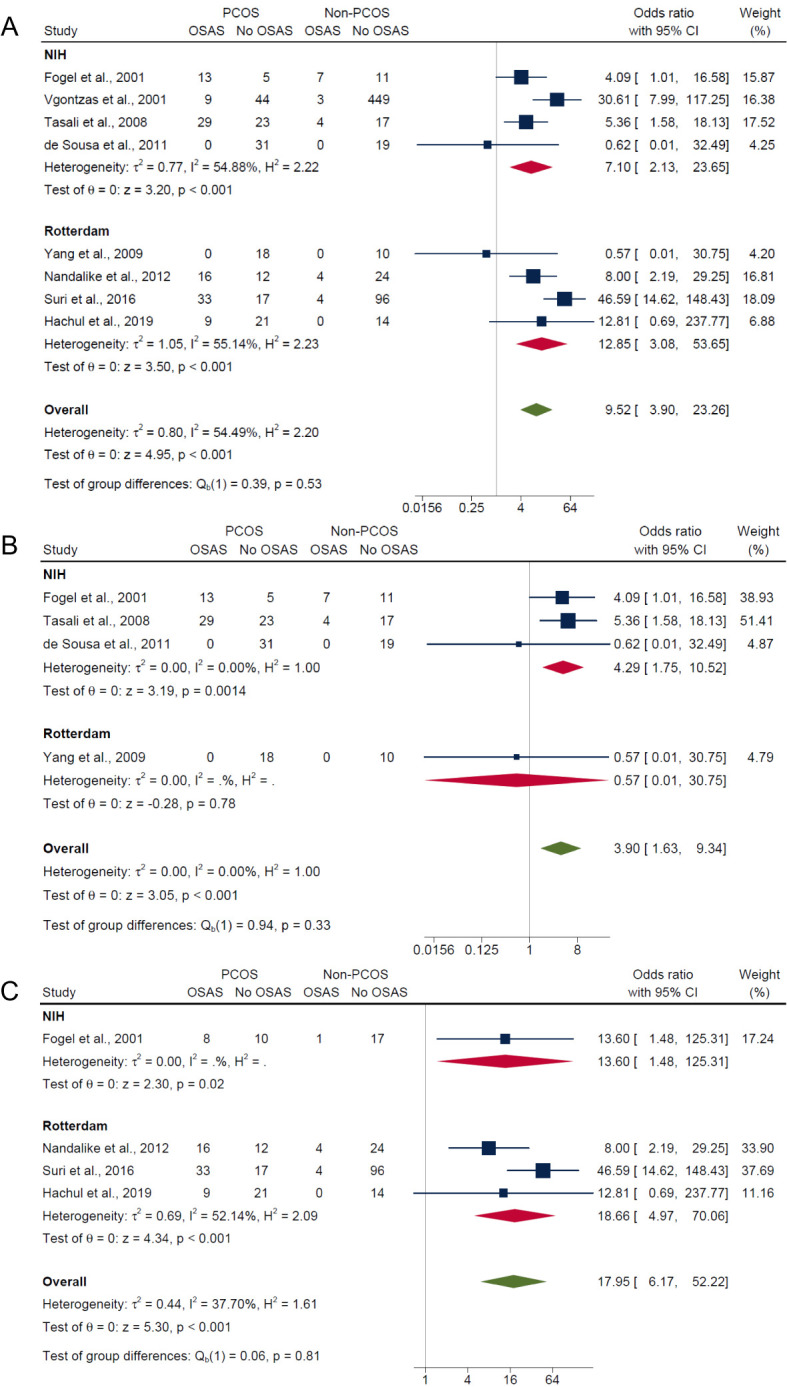
Subgroup analyses of symptom categories of OSAS in PCOS compared with non-PCOS by PCOS diagnostic criteria. **(A)** Composite OSA, **(B)** AHI≥5 only and **(C)** AHI≥5 with symptoms.

Independent of PCOS diagnostic criteria, results showed significantly higher risk of OSAS in PCOS compared with non-PCOS groups for composite OSA (Rotterdam criteria: OR: 12.85; 95% CI: 3.08 to 53.65; *P* < 0.001; 4 studies, n = 278 vs. NIH criteria: OR: 7.10; 95% CI: 2.13 to 23.65; *P* < 0.001; 4 studies, n = 664; **Figure 6A**) and AHI ≥5 with symptoms (Rotterdam criteria: OR: 18.66; 95% CI: 4.97 to 70.06; *P* < 0.001; 3 studies, n = 250 vs. NIH criteria: OR: 13.60; 95% CI: 1.48 to 125.31; *P* = 0.02; 1 study, n = 36; **Figure 6C**), but not for the AHI ≥5 only outcome where the risk was only higher in PCOS using the NIH criteria (Rotterdam criteria: OR: 0.57; 95% CI: 0.01 to 30.75; *P* = 0.78; 1 study, n = 28 vs. NIH criteria: OR: 4.29; 95% CI: 1.75 to 10.52; *P* = 0.0014; 3 studies, n = 159; **Figure 6B**).

#### 3.5.4 Subgroup analysis by ethnicity

**Figure 7:**
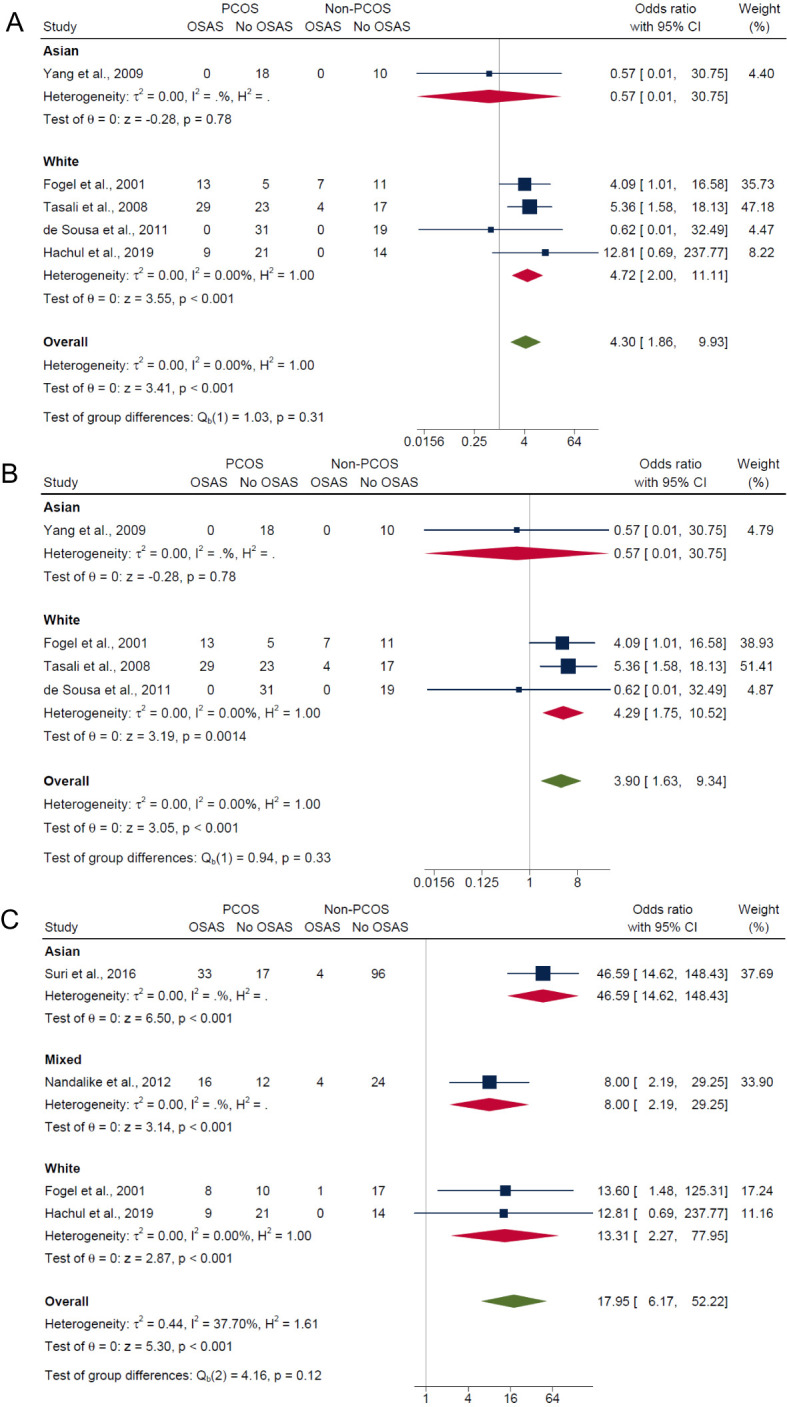
Subgroup analyses of symptom categories of OSAS in PCOS compared with non-PCOS by ethnicity. **(A)** Composite OSA, **(B)** AHI≥5 only and **(C)** AHI≥5 with symptoms.

Lastly, in the subgroup analysis by ethnicity, those of white ethnicity had significantly higher risk in PCOS compared with non-PCOS groups for composite OSA (OR: 4.72; 95% CI: 2.00 to 11.11; *P* < 0.001; 4 studies, n = 203; **Figure 7A**), AHI ≥5 only (OR: 4.29; 95% CI: 1.75 to 10.52; *P* = 0.0014; 3 studies, n = 159; **Figure 7B**) and AHI ≥5 with symptoms (OR: 13.31; 95% CI: 2.27 to 77.95; *P* < 0.001; 2 studies, n = 80; **Figure 7C**). In the Asian ethnicity subgroup with only one study, results were not significant for both composite OSA and AHI ≥5 only (ORs: 0.57; 95% CI: 0.01 to 30.75; *P* = 0.78; n = 28; **Figures 7A, B**), but significant for AHI ≥5 with symptoms (OR: 46.59; 95% CI: 14.62 to 148.43; *P* < 0.001; n = 150; **Figures 7C**). In the mixed ethnicity subgroup, there was significantly higher risk of OSAS in PCOS compared with non-PCOS groups for AHI ≥5 with symptoms (OR: 8.0; 95% CI: 2.19 to 29.25; *P* < 0.001; 1 study, n = 56; **Figure 7C**).

## 4 Discussion

This is the first comprehensive systematic review and meta-analysis to analyze the presence of OSAS and related symptoms in PCOS compared with non-PCOS populations using PSG/polygraph with or without validated sleep screening questionnaires. Our pooled analyses of eight studies totaling 942 participants showed that, overall, 37.0% of individuals with PCOS had OSA (29.0% adolescents; 40.0% adults), while only 6.0% without PCOS had OSA (7.0% adolescents; 7.0% adults). Moreover, those with PCOS were approximately 10 times more likely to have OSAS than those without PCOS. The risk of OSAS tended to be higher in those who had overweight or obesity, adult women and of white ethnicity compared with normal weight, adolescent and Asian populations respectively, but was largely independent of PCOS diagnostic criteria.

Compared to previous meta-analyses, the present study showed relatively higher pooled OSA prevalence (37.0% vs. 20.8-35.0%) and higher risk of OSAS (OR: 9.52 vs. ORs: 2.86-8.30) in women with PCOS relative to without PCOS (14, 22, 26). Differences in study methodology likely explain the varied estimates of OSAS risk compared with previous meta-analyses. For example, He et al. (26) included studies that screened for OSA using the Berlin questionnaire, studies that utilized PSG to diagnosed OSAS as well as studies that did not specify OSAS measurement. Inclusion of studies with objective, subjective and unknown measurements of OSAS could have underestimated the overall OSAS risk (OR: 2.86), as demonstrated in the present meta-analysis, where we report a markedly higher risk of OSAS (OR: 9.52) when including only those articles that employed formal sleep studies to assess OSAS.

The present meta-analysis reported, for the first time, a ~5-fold higher effect estimate for AHI≥5 with symptoms compared to AHI≥5 only outcomes among individuals with PCOS (OR: 17.95 vs. OR: 3.90) (15, 17–21, 40). This indicates that sleep disturbances and associated symptoms may drive the inflammation, oxidative stress and increased sympathetic excitability, plausibly alter the regulation of gonadotropins and gonadotropin-releasing hormones, thus contributing to the expression of pathophysiological features of PCOS such as hyperandrogenism and insulin resistance in a bidirectional association (41). Although treatment with continuous positive airway pressure (CPAP) therapy has shown to improve nocturnal apnea, daytime sleepiness, as well as metabolic outcomes for those with OSA, evidence regarding the impact of CPAP use in PCOS populations is lacking. The long-term cardiometabolic health benefits for treating OSA with CPAP are also not clearly established from RCTs, but research from observational studies seems to suggest a benefit on health outcomes from treatment in general populations, which may extend to those with PCOS (42). With a high proportion of the general population being minimally symptomatic, it is considered optimal to target and treat those with symptoms of OSAS such as non-restorative sleep, sleep-related complaints, daytime fatigue and sleepiness or hypertension. In light of this, targeted screening approaches for symptoms of OSAS, using questionnaires for example, have been recommended for high prevalence populations that include PCOS (43).

Similar to previous meta-analyses (22, 23, 26), our subgroup analysis demonstrated that the overall prevalence of OSA was higher in adults with PCOS, but not in adolescents with PCOS. This is expected, as PCOS is likely to precede the onset of OSA given that OSA may develop over time as features of PCOS like hyperandrogenism worsen, predisposing to OSA via effects on neural control of breathing and upper airway mechanics (44). Regarding differences by diagnostic criteria, we found significantly higher risks of OSAS in PCOS overall compared with those without PCOS, regardless of diagnostic criteria (45). Previous research has shown mixed results, with one meta-analysis reporting a greater proportion of OSAS in PCOS based on NIH criteria (26), while another reported no such variations across PCOS definitions (23). Consistent with He et al. (26), our subgroup analysis also demonstrated that the overall prevalence of OSAS was higher in those of white ethnicity with PCOS, but not in those of Asian ethnicity with PCOS. Finally, BMI variations were also evident in our pooled analyses, whereby overall risk of having OSAS in PCOS compared with non-PCOS was greater in those populations who were overweight/obese (OR: 7.62) and mixed weight (i.e., studies with sample populations that range across normal, overweight and obese) (OR: 39.09) compared with the normal weight (OR: 0.57) population. This finding is consistent with prior meta-analyses which have reported a higher risk of OSAS in individuals with both PCOS and obesity compared with those without obesity (23, 26). It should be noted that the very wide 95% confidence interval in the mixed weight group (95% CI: 13.31 to 114.77) imply imprecision in the overall certainty of the effect estimate. The normal weight group in our analysis also consisted of only Yang et al. (40), with a very small sample size (n = 28) and absence of AHI ≥5 in all participants. Of note, the BMI classification as per the World Health Organization (WHO) guideline (35) may not apply to Asian populations given that these were generated mostly from the White populations (46). Further, only two studies in our meta-analyses were from Asian populations (18, 40), thus this result should be interpreted accordingly.

PCOS may also be an independent contributor to the risk of OSAS. Our sensitivity analysis that was limited to studies that matched or controlled for confounders including BMI demonstrated a higher risk of OSAS in those with PCOS compared to non-PCOS (OR: 5.67). This finding suggests that other mechanistic factors besides weight such as hyperinsulinemia, hyperandrogenism and inflammation may also influence the OSAS-PCOS relationship (26). Insulin resistance and hyperinsulinemia, either directly or indirectly, through factors such as adiposity can lead to increase circulating androgen levels (6). The inflammatory and oxidative stress responses from recurrent episodes of apnea, coupled with cycles of hypoxia and reoxygenation in those with OSAS can also exacerbate the above pathophysiological features underpinning PCOS (41). It should be noted that despite prior meta-analysis reporting worse metabolic parameters (e.g., insulin resistance) in those with PCOS and OSAS compared to without OSAS (26), other common mechanistic factors like hyperandrogenism and insulin resistance linking PCOS and OSAS were not invariably measured independently of confounders like BMI. Many of their included studies were not included in our meta-analysis due to varying exclusion criteria (e.g., lack of PSG data) (16, 21). Further research is needed to understand the associations between pathophysiological risk factors of PCOS and the onset and progression of OSAS, including potential interactions with obesity.

To our knowledge, this meta-analysis is the first to comprehensively examine the prevalence and severity of OSAS and its related symptoms in PCOS. We included peer-reviewed published studies that utilized the PSG/polygraph tool along with related symptom assessments to ensure OSAS severity was well-defined and to minimize outcome detection bias. We followed rigorous, internationally endorsed methodology, guided by experts in the field as part of the 2023 PCOS Guideline update. We also assessed the certainty of the evidence at the outcome-level using the validated GRADE framework (33). Except for the composite OSA outcome where certainty of evidence was low, other symptom categories of OSAS outcomes (i.e., AHI≥5 only, AHI≥5 with symptoms, and AHI≥10 with symptoms) were deemed to have moderate certainty evidence. Similarly, publication bias assessments and rigorous quality and sensitivity analyses were conducted to enhance accuracy and minimize potential over- or under-estimation of results.

The main limitation of this review is that studies had a moderate to high risk of bias due to detection and selection biases. For example, some participants with PCOS were recruited from specialized clinics while healthy controls were drawn separately from the general population. The relatively small pooled sample sizes in this review (n = 280 PCOS and n = 662 non-PCOS) could have reduced the statistical power and generalizability of the results. Other limitations include, variable adjustment for important confounders such as age, BMI and ethnicity (16, 19, 20), the heterogenous BMI distribution of the study population, the varied definitions and assessment methods for OSA across studies that can lead to inconsistencies in reported outcomes. In addition, under-representation of ethnic and geographic groups (e.g., Asian or mixed-ethnicity) can restrict the generalizability of the findings. Finally, the heterogeneity in OSAS prevalence estimates (e.g., *I*² = 96.8% for OSAS in PCOS) signify variability population characteristics. Hence, the findings should be interpreted in view of these points. Nevertheless, the impact of the included studies is likely to be minimal, since the exclusion of these studies in sensitivity analysis did not influence the overall meta-analysis results. Due to the limited studies, single-paper analyses as well as subgroup analyses where only one study belonged to the comparison group should be interpreted with caution as this may lead to chance findings. Studies exploring PCOS population subgroups with varying symptomatic OSAS profiles are warranted, as are studies exploring ethnically diverse and adolescent populations, to enhance generalization to the global PCOS population. These are crucial evidence gaps as morbidity of OSAS is related to age and racial differences in both clinical and metabolic profiles of PCOS.

## 5 Conclusion

The present systematic review and meta-analysis found that, independent of PCOS diagnostic criteria, the overall risk of OSAS is markedly higher in PCOS compared with non-PCOS populations, especially in adult women, those with overweight/obesity and of white ethnicity, and was more pronounced with increasing symptomatic OSAS severity. Using the methodology from the 2023 International Evidence-based PCOS Guideline, our findings recommend that women with PCOS be screened for symptoms of OSAS using validated questionnaires combined with a thorough sleep apnea history to target OSA treatment to those more likely to benefit (1). Recognizing the heightened risk of OSAS in PCOS will aid in identifying those most susceptible, to enable the development of more effective and targeted strategies for OSAS treatment.
