# Supplementary material for: Obstructive sleep apnea syndrome in polycystic ovary syndrome: a systematic review and meta-analysis
Source: Front Endocrinol (Lausanne). 2025 Apr 4;16:1532519. doi: 10.3389/fendo.2025.1532519 (PMC12006010; doi:10.3389/fendo.2025.1532519)
Supplement: Supplementary file 1 [file Image1.pdf]

A.

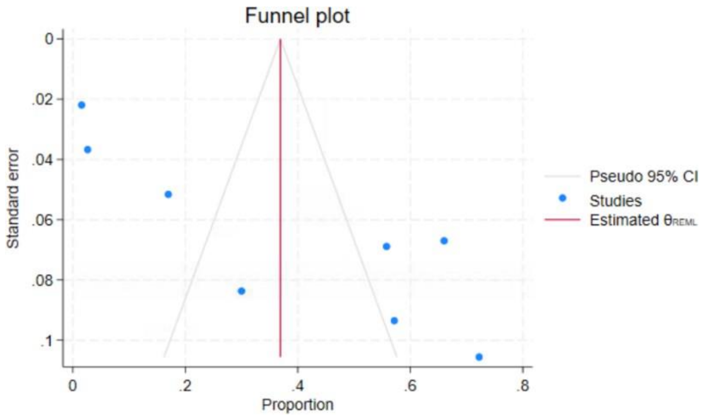

Visual asymmetry in funnel plots showed small study effects.

B.

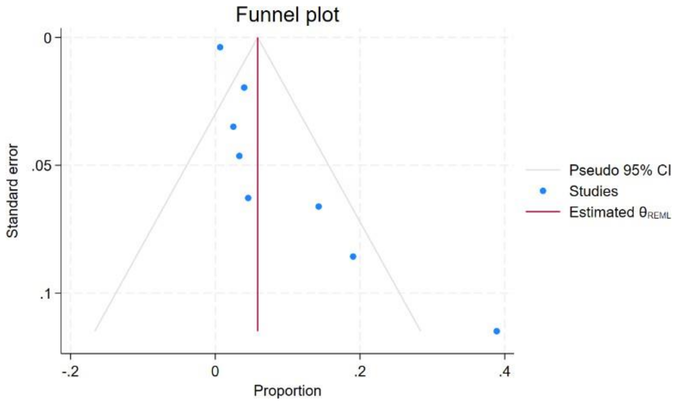

Visual asymmetry in funnel plots showed small study effects.

Supplementary Figure 1. Funnel plots to assess for publication bias for overall prevalence of OSA. (A) PCOS population and (B) non-PCOS population.
