# Supplementary material for: Obstructive sleep apnea syndrome in polycystic ovary syndrome: a systematic review and meta-analysis
Source: Front Endocrinol (Lausanne). 2025 Apr 4;16:1532519. doi: 10.3389/fendo.2025.1532519 (PMC12006010; doi:10.3389/fendo.2025.1532519)
Supplement: Supplementary file 2 [file Image2.pdf]

A.

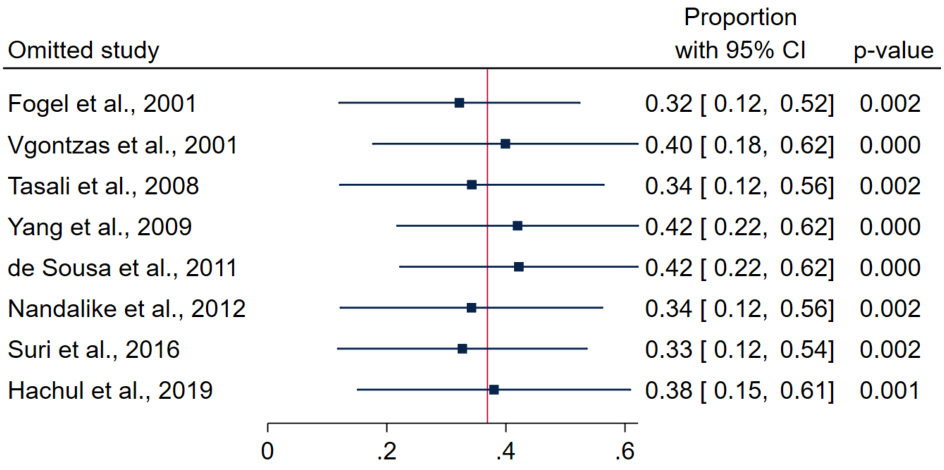

Results remained significant. No individual study showed influence in the overall results.

B.

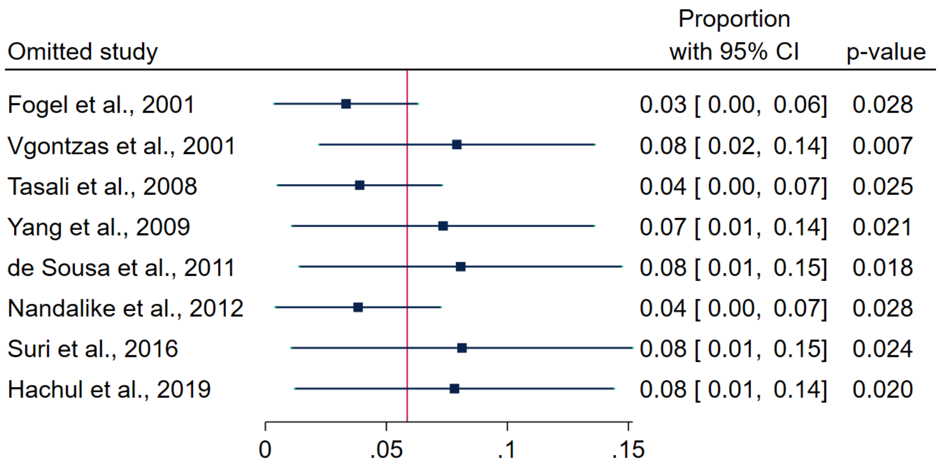

Results remained significant. No individual study showed influence in the overall results.

Supplementary Figure 2. Sensitivity analysis (leave-one-out) for prevalence of OSA. (A) PCOS population and (B) non-PCOS population.
