# Supplementary material for: Obstructive sleep apnea syndrome in polycystic ovary syndrome: a systematic review and meta-analysis
Source: Front Endocrinol (Lausanne). 2025 Apr 4;16:1532519. doi: 10.3389/fendo.2025.1532519 (PMC12006010; doi:10.3389/fendo.2025.1532519)
Supplement: Supplementary file 3 [file Image3.pdf]

A.

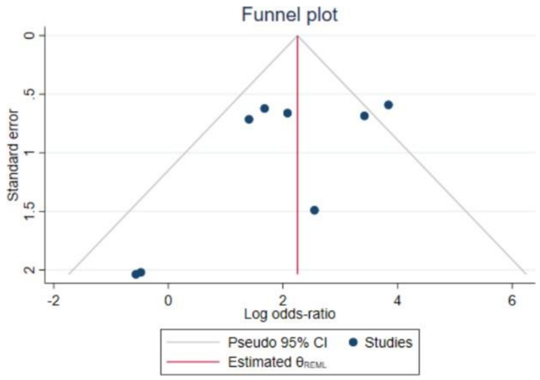

Visual symmetry in funnel plots showed no small study effects.

B.

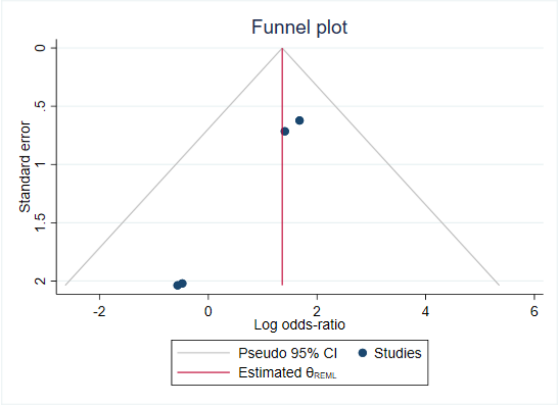

Visual symmetry in funnel plots showed no small study effects.

C.

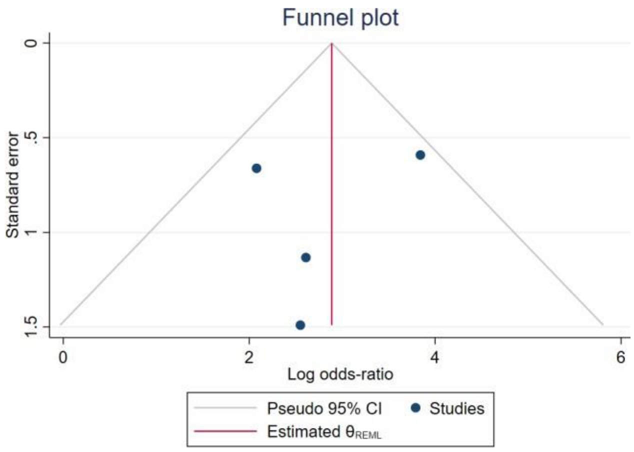

Visual symmetry in funnel plots showed no small study effects.

Supplementary Figure 3. Funnel plots to assess for publication bias for symptom categories of OSAS in PCOS vs. non-PCOS populations. (A) Composite OSA, (B) AHI≥5 only and (C) AHI≥5 with sleep-related symptoms.
