# Supplementary material for: Obstructive sleep apnea syndrome in polycystic ovary syndrome: a systematic review and meta-analysis
Source: Front Endocrinol (Lausanne). 2025 Apr 4;16:1532519. doi: 10.3389/fendo.2025.1532519 (PMC12006010; doi:10.3389/fendo.2025.1532519)
Supplement: Supplementary file 4 [file Image4.pdf]

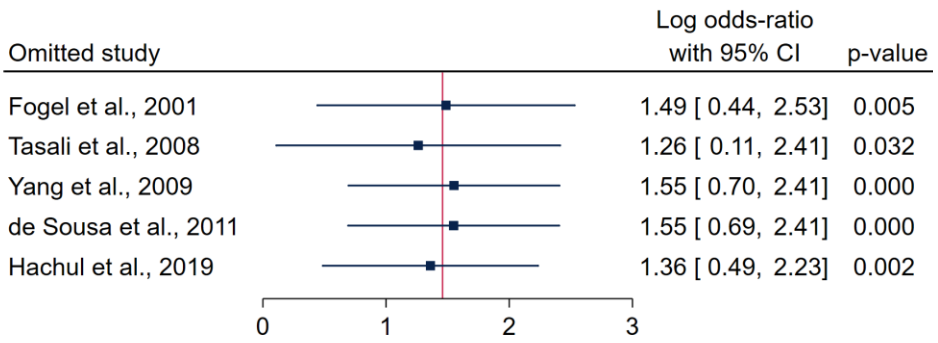

Results remained significant. No individual study showed influence in the overall results.

Supplementary Figure 4. Sensitivity analysis (leave-one-out) for composite OSA.
