# Supplementary material for: Obstructive sleep apnea syndrome in polycystic ovary syndrome: a systematic review and meta-analysis
Source: Front Endocrinol (Lausanne). 2025 Apr 4;16:1532519. doi: 10.3389/fendo.2025.1532519 (PMC12006010; doi:10.3389/fendo.2025.1532519)
Supplement: Supplementary file 5 [file Image5.pdf]

A.

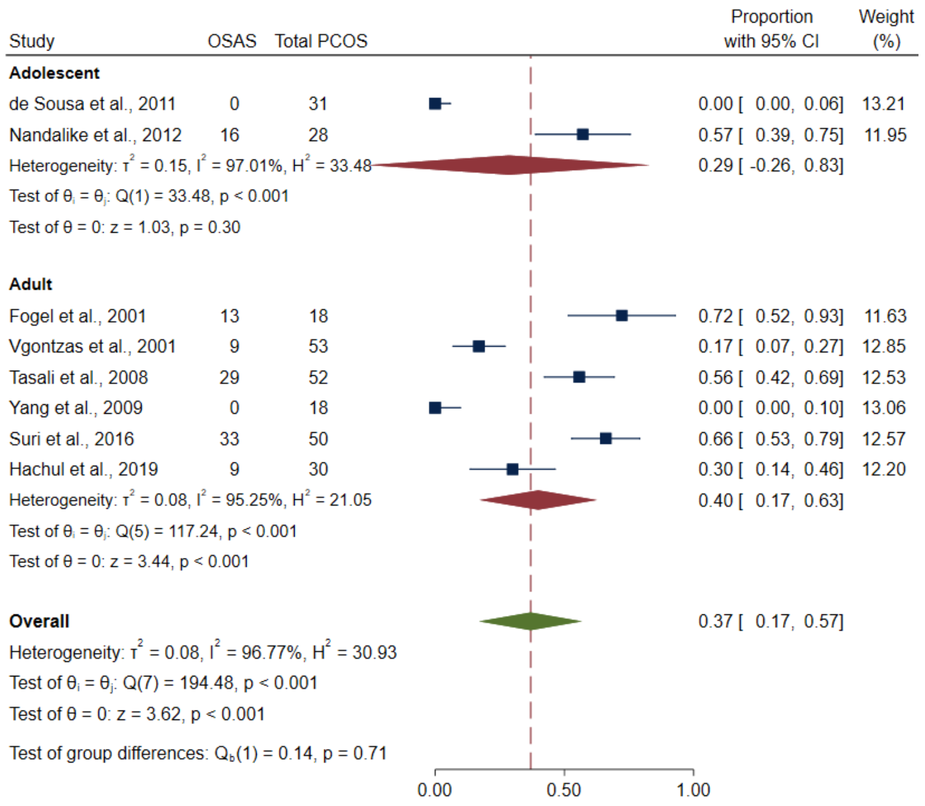

B.

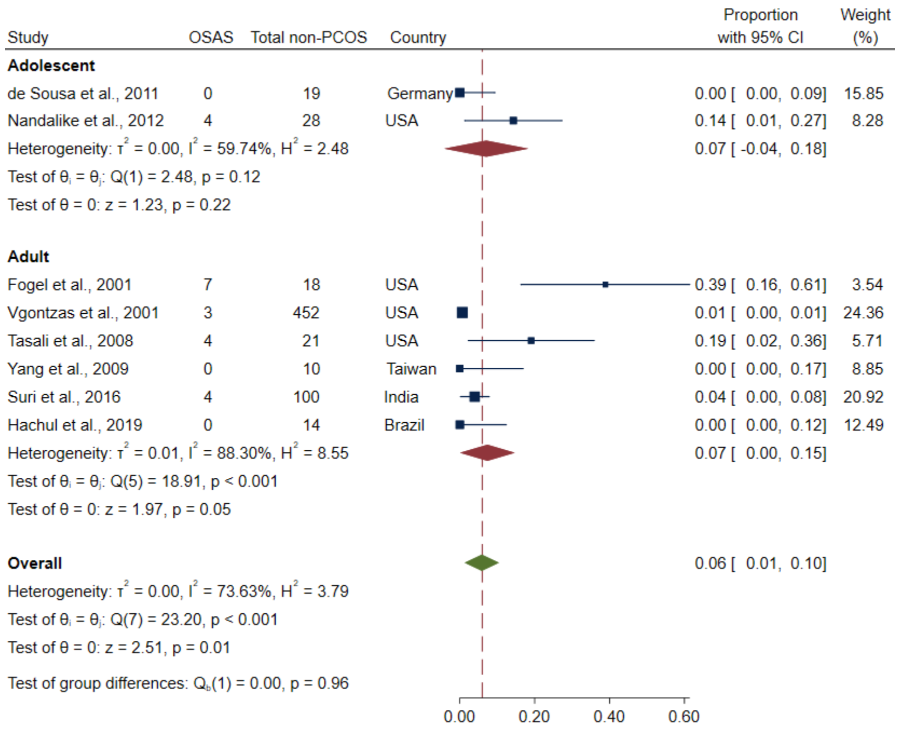

Supplementary Figure 5. Prevalence of OSA by age subgroup. (A) PCOS population and (B) non-PCOS population.
