# Supplementary material for: Obstructive sleep apnea syndrome in polycystic ovary syndrome: a systematic review and meta-analysis
Source: Front Endocrinol (Lausanne). 2025 Apr 4;16:1532519. doi: 10.3389/fendo.2025.1532519 (PMC12006010; doi:10.3389/fendo.2025.1532519)
Supplement: Supplementary file 6 [file Table1.docx]

| **Supplementary Table 1.** Eligibility criteria (PECOS) for study inclusion. | | | | |  |
| --- | --- | --- | --- | --- | --- |
|  | **Participants (P)** | **Exposure (E)** | **Comparator (C)** | **Outcome (O)** | **Study design (S)** |
| **INCLUSION** | Females of any age, ethnicity, weight and comorbidity (e.g., infertility) | Females with a diagnosis of any phenotype of PCOS (diagnosed by Rotterdam, NIH or AES) | Females without  PCOS | Sleep apnea on formal sleep studies:  Level 1, in-lab,  Level 2, same set up in home or  Level 3, ambulatory limited channel  Polysomnography (PSG)  Prevalence and severity of obstructive  sleep apnea (including obstructive sleep apnea, sleep apnea, sleep apnoea, sleep-disordered breathing, snoring) on  1. Apnea hypopnea index (AHI) or respiratory disturbance index (RDI) >5 events per hour and  2. Sleep apnea syndrome >5 and symptoms  Severity by AHI diagnostic categories:  Mild: 5-14  Moderate: >15-29  Severe: >30 | Cohort studies (e.g., case-control, controlled cross-sectional) and randomized controlled trial (RCT) studies.  Included studies were not restricted by language and year of publication.  Other systematic reviews were included initially for screening references to identify additional eligible studies. |
| **EXCLUSION** | Studies of other  population groups | Studies with self-reported  PCOS diagnosis | Studies without a control or  comparison arm | Studies without clinical outcomes (mechanistic studies);  Studies not reporting validated sleep outcomes;  Studies describing sleep apnea symptoms through questionnaires without formal measurement;  Studies with self-reported or doctor diagnosed OSA without formal measurement |  |
| Abbreviations: AES, Androgen Excess Society; AHI, apnea hypopnea index; NIH, National Institute of health; PCOS, polycystic ovary syndrome; PSG, polysomnography; RDI, respiratory distress index. | | | | | |
