# Supplementary material for: Obstructive sleep apnea syndrome in polycystic ovary syndrome: a systematic review and meta-analysis
Source: Front Endocrinol (Lausanne). 2025 Apr 4;16:1532519. doi: 10.3389/fendo.2025.1532519 (PMC12006010; doi:10.3389/fendo.2025.1532519)
Supplement: Supplementary file 7 [file Table2.docx]

| **Supplementary Table 2.** Search strategies for electronic database searches as of 16^th^ May 2024. | |
| --- | --- |
| **OVID Medline, EBM reviews, PsycInfo, Embase** | **CINAHL** |
| **# Query**  1 exp polycystic ovary syndrome/  2 polycystic ovar*.mp.  3 poly-cystic ovar*.mp.  4 PCO*.mp.  5 (stein-leventhal or leventhal).mp.  6 anovulation/  7 anovulat*.mp.  8 oligo-ovulat*.mp.  9 oligoovulat*.mp.  10  (ovar* adj5 (sclerocystic or polycystic or poly-cystic or  degenerat* or hyperandrogen* or hyper-androgen*)).mp.  11 1 or 2 or 3 or 4 or 5 or 6 or 7 or 8 or 9 or 10  12 exp Sleep Apnea, Obstructive/  13 exp Sleep Apnea Syndromes/  14 exp Obesity Hypoventilation Syndrome/  15 exp Sleep Apnea, Central/  16 exp Polysomnography/  17 exp Snoring/  18 sleep apn*.mp.  19 apn*-hypopn*.mp.  20 sleep hypopn*.mp.  21 sleep hypoventilation.mp.  22 obesity hypoventilation.mp.  23 apnoea.mp.  24 apnea.mp.  25 apneic.mp.  26 apnoeic.mp.  27 hypopneic.mp.  28 hypopnoeic.mp.  29 sleep disordered breathing.mp.  30 sleep-disordered breathing.mp.  31 (sleep and breathing).mp.  32 (sleep and respiratory).mp.  33 upper-airway resistance.mp.  34 upper airway resistance.mp.  35  (sleep adj5 (apn* or hypopn* or hypoventilation or breathing or  respiratory)).mp.  36 polysomnography.mp.  37 snoring.mp.  38 snore.mp.  39 SDB.mp.  40 OSA.mp.  41 OSAS.mp.  42 SAHS.mp.  43 OSAHS.mp.  44 SAS.mp.  45 SHS.mp.  46 pickwickian.mp.  47  12 or 13 or 14 or 15 or 16 or 17 or 18 or 19 or 20 or 21 or 22 or  23 or 24 or 25 or 26 or 27 or 28 or 29 or 30 or 31 or 32 or 33 or  34 or 35 or 36 or 37 or 38 or 39 or 40 or 41 or 42 or 43 or 44 or  45 or 46  48 11 and 47 | **# Query**  1 SU polycystic ovary syndrome  2 "polycystic ovar*"  3 "poly-cystic ovar*"  4 "PCO*"  5 "stein-leventhal or leventhal"  6 SU anovulation  7 SU ovarian cysts  8 "anovulat*"  9 "oligo-ovulat*"  10 "oligoovulat*"  11  "ovar* N5 sclerocystic or ovar* N5  polycystic or ovar* N5 poly-cystic or  ovar* N5 degenerat* or ovar* N5  hyperandrogen* or ovar* N5  hyperandrogen*"  12  S1 OR S2 OR S3 OR S4 OR S5 OR  S6 OR S7 OR S8 OR S9 OR S10  OR S11  13 SU Sleep Apnea, Obstructive  14 SU Sleep Apnea Syndromes  15  SU Obesity Hypoventilation  Syndrome  16 SU Sleep Apnea, Central  17 SU Polysomnography  18 SU Snoring  19 "sleep apn*"  20 "apn*-hypopn*"  21 "sleep hypopn*"  22 "sleep hypoventilation"  23 "obesity hypoventilation"  24 "apnoea"  25 "apnea"  26 "apneic"  27 "apnoeic"  28 "hypopneic"  29 "hypopnoeic"  30 "sleep disordered breathing"  31 "sleep-disordered breathing"  32 "sleep and breathing"  33 "sleep and respiratory"  34 "upper-airway resistance"  35 "upper airway resistance"  36  "sleep N5 apn* or sleep N5 hypopn*  or sleep N5 hypoventilation or sleep  N5 breathing or sleep N5 respiratory"  37 "polysomnography"  38 "snoring"  39 "snore"  40 "SDB"  41 "OSA"  42 "OSAS"  43 "SAHS"  44 "OSAHS"  45 "SAS"  46 "SHS"  47 "pickwickian"  48  S13 OR S14 OR S15 OR S16 OR  S17 OR S18 OR S19 OR S20 OR  S21 OR S22 OR S23 OR S24 OR  S25 OR S26 OR S27 OR S28 OR  S29 OR S30 OR S31 OR S32 OR  S33 OR S34 OR S35 OR S36 OR  S37 OR S38 OR S39 OR S40 OR  S41 OR S42 OR S43 OR S44 OT  S45 OR S46 OR S47  49 S12 AND S48 |
