# Supplementary material for: Obstructive sleep apnea syndrome in polycystic ovary syndrome: a systematic review and meta-analysis
Source: Front Endocrinol (Lausanne). 2025 Apr 4;16:1532519. doi: 10.3389/fendo.2025.1532519 (PMC12006010; doi:10.3389/fendo.2025.1532519)
Supplement: Supplementary file 8 [file Table3.docx]

| **Supplementary Table 3.** Reasons for study exclusion. | | |
| --- | --- | --- |
| **#** | **Reference** | **Reason** |
| 1 | Teede, Helena J., et al. "Recommendations from the 2023 international evidence-based guideline for the assessment and management of polycystic ovary syndrome." European journal of endocrinology 189.2 (2023): G43-G64. | Guideline |
| 2 | Kumarendran, Balachandran, et al. "Increased risk of obstructive sleep apnoea in women with polycystic ovary syndrome: a population-based cohort study." European journal of endocrinology 180.4 (2019): 265-272.. | Study outcome - self-reported  or doctor diagnosed |
| 3 | Kahal, Hassan, et al. "The association between obstructive sleep apnea and metabolic abnormalities in women with polycystic ovary syndrome: a systematic review and meta-analysis." Sleep 41.7 (2018): zsy085. | Review |
| 4 | Lin, Ting-Yang, et al. "Risk of developing obstructive sleep apnea among women with polycystic ovarian syndrome: a nationwide longitudinal follow-up study." Sleep Medicine 36 (2017): 165-169. | Study outcome - self-reported  or doctor diagnosed without validation |
| 5 | Chatterjee, Bidisha, et al. "Impact of sleep-disordered breathing on metabolic dysfunctions in patients with polycystic ovary syndrome." Sleep medicine 15.12 (2014): 1547-1553. | Comparator – no control group |
| 6 | Sirmans, Susan M., et al. "Epidemiology and comorbidities of polycystic ovary syndrome in an indigent population." Journal of Investigative Medicine 62.6 (2014): 868-874. | Study outcome - self-reported  or doctor diagnosed without  validation |
| 7 | de Sousa, Gideon, et al. "The impact of insulin resistance and hyperandrogenemia on polysomnographic variables in obese adolescents with polycystic ovarian syndrome." Sleep and Breathing 16 (2012): 169-175. | Study outcome - only reporting  mean (SD) of AHI /obstructive  apnea events |
| 8 | Sanders, Mark H. "Increased risk of obstructive sleep apnea in obese women with polycystic ovary syndrome (a review of two related articles). | Review |
| 9 | Dunaif, Andrea. "Genes, aging and sleep apnea in polycystic ovary syndrome." Nature Reviews Endocrinology 8.2 (2012): 72-74. | Review |
| 10 | Nandalike, Kiran, et al. "Screening for sleep-disordered breathing and excessive daytime sleepiness in adolescent girls with polycystic ovarian syndrome." The Journal of pediatrics 159.4 (2011): 591-596. | Study outcome - through  questionnaire without formal  assessment |
| 11 | de Sousa, Gideon, et al. "A comparison of polysomnographic variables between adolescents with polycystic ovarian syndrome with and without the metabolic syndrome." Metabolic syndrome and related disorders 9.3 (2011): 191-196. | Study outcome - only reporting  mean (SD) of AHI /obstructive  apnoea events |
| 12 | Sousa, Gideon de, et al. "A comparison of polysomnographic variables between adolescents with polycystic ovarian syndrome and healthy controls." International Journal of Clinical Medicine 1.02 (2010): 48-53. | Study outcome - only reporting  mean (SD) of AHI /obstructive  apnoea events |
| 13 | Tasali, Esra, Eve Van Cauter, and David A. Ehrmann. "Relationships between sleep disordered breathing and glucose metabolism in polycystic ovary syndrome." The Journal of Clinical Endocrinology & Metabolism 91.1 (2006): 36-42. | Comparator – no control group |
| 14 | Underland, Lisa, et al. "3199 Effect of OSAS on Insulin Sensitivity and Cardiovascular Risk in PCOS Adolescents." Journal of Clinical and Translational Science 3.s1 (2019): 39-39. | Conference proceeding |
| 15 | Thannickal, Aneesa, et al. "Eating, sleeping and sexual function disorders in women with polycystic ovary syndrome (PCOS): A systematic review and meta‐analysis." Clinical endocrinology 92.4 (2020): 338-349. | Review |
| 16 | Underland, Lisa J., et al. "Insulin sensitivity and obstructive sleep apnea in adolescents with polycystic ovary syndrome." Minerva Endocrinology (2022). | Comparator – OSA not assessed in control group |
| 17 | Xerfan, Ellen MS, et al. "Polycystic ovary syndrome and its possible association with sleep complaints: PCOS and Sleep." Archives of Women's Mental Health 24.6 (2021): 1055-1057. | Commentary |
| 18 | Zevin, Erika L., and M. Tracy Bekx. "50. Positive Screen for Sleep Apnea in Adolescents With Polycystic Ovary Syndrome Is Independent of BMI and Metabolic Risk." Journal of Pediatric and Adolescent Gynecology 32.2 (2019): 214. | Conference proceeding |
| 19 | Saha, S., et al. "Sleep disorders in polycystic ovary syndrome (PCOS) and their metabolic correlates." American Thoracic Society 2018. A2415-A2415. | Conference proceeding |
| 20 | Hachul, H., et al. "Sleep disorders in women with polycystic ovary syndrome: the influence of obesity and hyperandrogenism." Abstracts/Sleep Medicine 40.e3ee185 (2017): e125. | Conference proceeding |
| 21 | Zea-Hernandez, Johanna, et al. "A30 Pediatric sleep and sleep medicine: Sleep Disordered Breathing In Adolescent Girls With Polycystic Ovary Syndrome." American Journal of Respiratory and Critical Care Medicine 189 (2014): 1. | Conference proceeding |
| 22 | Abdel Wahab, Nashwa H., et al. "Sleep related breathing disorders in Egyptian females with polycystic ovary syndrome." American Thoracic Society, 2013. A3447-A3447. | Conference proceeding |
| 23 | Temple, K. A., et al. "Adiponectin levels in obese women with and without PCOS: Impact of obstructive sleep apnea." 95th Annual Meeting and Expo of the Endocrine Society, ENDO. 2013. | Conference proceeding |
| 24 | Morselli, L. L., et al. "Effects of polycystic ovary syndrome (PCOS) on REM and non-REM sleep in African-American (AA) women." 95th Annual Meeting and Expo of the Endocrine Society, ENDO, San Francisco, CA United States. 2013. | Conference proceeding |
| 25 | Temple, Karla A., et al. "Abnormal glucose tolerance in women with polycystic ovary syndrome (PCOS): role of sex steroids and obstructive sleep apnea." Diabetes. Vol. 62. | Conference proceeding |
| 26 | Hachul, H., et al. "Polycystic ovary syndrome: A comparative study of sleep parameters in patients with and without hyperandrogenemia." SLEEP. Vol. 35. | Conference proceeding |
| 27 | Yang HsiaoPing, Yang HsiaoPing, et al. "A pilot study of heart rate variability and apneic-hypopneic events in non-obese women with polycystic ovary syndrome during sleep." (2010): 9-21. | Study outcome - only reporting  mean (SD) of AHI /obstructive  apnoea events |
| 28 | Gopal, Mira, et al. "The role of obesity in the increased prevalence of obstructive sleep apnea syndrome in patients with polycystic ovarian syndrome." Sleep medicine 3.5 (2002): 401-404. | Comparator – no control group |
| 29 | Kahal, Hassan, et al. "The prevalence of obstructive sleep apnoea in women with polycystic ovary syndrome: a systematic review and meta-analysis." Sleep and Breathing 24 (2020): 339-350. | Review |
| 30 | Zhang, Jiayu, et al. "Sleep disturbances, sleep quality, and cardiovascular risk factors in women with polycystic ovary syndrome: systematic review and meta-analysis." Frontiers in Endocrinology 13 (2022): 971604. | Review |
| 31 | Wang, Chaoyu, et al. "A meta-analysis of the relationship between polycystic ovary syndrome and sleep disturbances risk." Frontiers in Physiology 13 (2022): 957112. | Review |
| 32 | Helvaci, Nafiye, et al. "Polycystic ovary syndrome and the risk of obstructive sleep apnea: a meta-analysis and review of the literature." Endocrine Connections 6.7 (2017): 437-445. | Review |
