# Supplementary material for: Obstructive sleep apnea syndrome in polycystic ovary syndrome: a systematic review and meta-analysis
Source: Front Endocrinol (Lausanne). 2025 Apr 4;16:1532519. doi: 10.3389/fendo.2025.1532519 (PMC12006010; doi:10.3389/fendo.2025.1532519)
Supplement: Supplementary file 9 [file Table4.docx]

| **Supplementary Table 4.** Study characteristics and quality appraisal templates: cross-sectional or case-control studies. | | |
| --- | --- | --- |
| **Study ID** | de Sousa et al. 2011 | |
| **Study Citation** | de Sousa G, Schlüter B, Menke T, Trowitzsch E, Andler W, Reinehr T. Relationships between polysomnographic variables, parameters of glucose metabolism, and serum androgens in obese adolescents with polycystic ovarian syndrome. J Sleep Res. 2011 Sep;20(3):472-8. doi: 10.1111/j.1365-2869.2010.00902.x | |
| **Study country** | Germany | |
| **Characteristics and external validity – is this study and its results generalizable to my systematic review question?** | | |
| **Patient/population/ participants** | 31 obese adolescents aged 13–16 years with PCOS (mean age 15.0 years ± 1.0, mean BMI 32.7 kg/m^2^ ± 6.2, mean SDS–BMI 2.5 ± 0.8). The study group consisted of 13 new subjects and 18 others who had been part of our previous study group (de Sousa et al., 2010). Recruited form the outpatient Obesity and Endocrine Department of the Vestische Children’s Hospital, Datteln, Germany. | |
| **Control population** | 19 healthy obese adolescents without PCOS aged 13–17 years (mean age 15.2 years ± 1.1, mean BMI 32.4 kg/m^2^ ± 4.0, mean SDS–BMI 2.5 ± 0.5). Recruited from the outpatient Obesity and Endocrine Department of the Vestische Children’s Hospital, Datteln, Germany. | |
| **PCOS diagnostic criteria** | NIH 1992; Conditions such as non-classical adrenal 21-hydroxylase deficiency, androgen secreting  tumours and Cushing’s syndrome were excluded by appropriate tests before the diagnosis of PCOS was made. | |
| **N per group** | PCOS group – 31  Non-PCOS group – 19 | |
| **Setting** | Hospital outpatient | |
| **Outcomes (primary and other) with definition/tool (eg. self-reported, fasting etc.)** | Primary outcomes:  ‐ Obstructive sleep apnoea syndrome (AHI ≥5) [n (%)]  Outcomes not relevant:  ‐ Apnoea index [mean (SD)]  -Hypopnoea index [mean (SD)]  -Apnoea – hypopnoea index [mean (SD)]  -Absolute number of obstructive apnoea [mean (SD)]  -Sleep Stage 1 (%) [mean (SD)]  -Sleep Stage 2 (%) [mean (SD)]  -Sleep Stage 3 and 4 (%) [mean (SD)]  - REM sleep (%) [mean (SD)]  - Time in bed (min) [mean (SD)]  -Total sleep time [mean (SD)]  -Sleep efficiency (%) [mean (SD)]  -Sleep onset latency (min) [mean (SD)]  -Total wake time (TWT) (min) [mean (SD)]  -Wakefulness after sleep onset (WASO) [mean (SD)] | |
| **Does the study have a clearly focused question and/or PICO?** | Yes  Partial  No  Not reported | Yes  The aim of this study was to compare polysomnographic variables of obese adolescents with polycystic ovarian syndrome (PCOS) to those of healthy controls. |
| **Inclusion criteria** | Yes  Partial  No  Not reported | Partial  Partial generalisability as limited to obese adolescents aged 13–16 years. |
| **Exclusion criteria** | Yes  Partial  No  Not reported | Yes  Conditions such as non-classical adrenal 21-hydroxylase deficiency, androgen-secreting tumours and Cushing’s syndrome were excluded by appropriate tests before the diagnosis of PCOS was made. The control patients were all healthy and had no  history pointing towards sleep-related breathing disorders. All control patients had normal menstrual cycles (28–35 days) and no clinical signs of androgen excess, thereby excluding PCOS by NIH definition (Zawadski and Dunaif, 1992). All participants were without evidence of other diseases, including conditions which are common causes of apnoea in children and adolescents, such as adenotonsillar hypertrophy, craniofacial abnormalities or neuromuscular disease. Furthermore, all participants were not currently taking any medications. |
| **If there were specified inclusion/exclusion criteria, were these appropriate?** | Yes  Partial  No  Not reported | Yes |
| **Is a cross sectional or case-control study the appropriate design to answer this question?** | Yes  Partial  No  Not reported | Yes  This study is cross-sectional. |
| **Was there sufficient duration of follow-up for outcomes to occur?** | Yes  Partial  No  Not reported | Not relevant (the study is cross-sectional in nature). |
| **Was matching performed?** | Yes  Partial  No  Not reported | No |
| Summary Result/s | PCOS was not associated with respiratory polysomnographic variables. | |
| **Internal validity – has this study been conducted rigorously in order to reduce bias?** | | |
| **Selection bias** | | |
| **Were the cases and controls taken from comparable populations?** | Yes  Partial  No  Not reported | Yes  Both recruited from outpatient Obesity and Endocrine Department of the Vestische Children’s Hospital. |
| **Was the case definition adequate and established in a standard, valid, and**  **reliable way?** | Yes  Partial  No  Not reported | Yes  The diagnosis of PCOS was based on the definition of the NIH (Zawadski and Dunaif, 1992). Conditions such as non-classical adrenal 21-hydroxylase deficiency, androgen-secreting tumours and Cushing’s syndrome were excluded by appropriate tests before the diagnosis of PCOS was made. |
| **Was the control status established in a standard, valid and reliable way?** | Yes  Partial  No  Not reported | Yes  The control patients were all healthy and had no history pointing towards sleep-related breathing disorders. All control patients had normal menstrual cycles (28–35 days) and no clinical signs of androgen excess, thereby excluding PCOS by NIH definition  (Zawadski and Dunaif, 1992). |
| **Performance bias** | | |
| **Aside from the exposure/intervention, were the groups treated the same?** | Yes  Partial  No  Not reported | Yes  Menstrual history was obtained from all the patients. All patients underwent physical examination. Percentage body fat was calculated in all participants. Serum insulin and glucose concentrations were measured in all participants in the fasting status. An oral glucose tolerance test (OGTT) was performed in all girls. All girls underwent  overnight 12-channel polysomnography. |
| **Detection bias** | | |
| **Were measurements (for exposures or outcomes) carried out and calculated in a standard, valid and reliable way?** | Yes  Partial  No  Not reported | Yes  Menstrual history was obtained from all the patients. All patients underwent physical examination. All girls underwent overnight 12-channel polysomnography. |
| **Were outcome assessors blind to case and control status?** | Yes  Partial  No  Not reported | Not reported |
| **Were all outcomes measured in a standard, valid and reliable way?** | Yes  Partial  No  Not reported | Yes  All girls underwent overnight 12-channel polysomnography. |
| **Were outcomes assessed objectively and**  **independently?** | Yes  Partial  No  Not reported | Yes  All polysomnographic records were evaluated by experienced paediatric somnologists (B. S. and D. B.). Sleep was staged according to standard criteria (Rechtschaffen and Kales, 1968). Arousals and respiratory events were defined according to the guidelines of the American Academy of Sleep Medicine (AASM, 1992, 1999). |
| **Attrition bias** | | |
| **What percentage of the individuals recruited into each arm of the study were lost to follow up?** | Yes  Partial  No  Not reported | Not relevant to cross‐sectional study. |
| **What percentage of the individuals were not included in the analysis?** | Yes  Partial  No  Not reported | Not reported |
| **Report bias** | | |
| **Is the paper free of selective outcome reporting?** | Yes  Partial  No  Not reported | Not reported  No protocol or PROSPERO. |
| **Confounding** | | |
| **Are the cohorts comparable on the basis of design or analysis?** | Yes  Partial  No  Not reported | Yes  The girls in the study group and in the control group did not differ significantly in respect of age (P = 0.46), weight in kg (P = 0.70), BMI (P = 0.84), SDS–BMI (P = 0.74), waist circumference (P = 0.51), fasting glucose (P = 0.13), fasting insulin (P = 0.26), HOMA (P = 0.38), QUICKI (P = 0.95), 2-h glucose in oGTT (P = 0.08), serum dehydroepiandrosterone sulphate (P = 0.52) and serum androstendione (P = 0.08). Total testosterone was significantly higher in the girls with PCOS (P = 0.04) – but this is related to PCOS. |
| **Other bias** | | |
| **Were there any conflicts of interest in the writing or funding of this study?** | Yes  Partial  No  Not reported | No |
| **Was the study sufficiently powered to detect any differences between the groups?** | Yes  Partial  No  Not reported | Not reported |
| **If statistical analysis was undertaken, was this appropriate?** | Yes  Partial  No  Not reported | Yes  Comparisons between the cases and controls were performed by t-test for unpaired observations. |
| **Comments** | Small sample size | |
| **What is the overall risk of bias?** | Low  Moderate  High Insufficient  information | Moderate |
| **Did risk of bias differ by outcome (eg.**  **primary outcome was low risk but rest**  **were high)?** | No | |

| **Study ID** | Fogel et al. 2001 | |
| --- | --- | --- |
| **Study Citation** | Fogel RB, Malhotra A, Pillar G, Pittman SD, Dunaif A, White DP. Increased prevalence of  obstructive sleep apnea syndrome in obese women with polycystic ovary syndrome. J Clin  Endocrinol Metab. 2001 Mar;86(3):1175-80. doi: 10.1210/jcem.86.3.7316 | |
| **Study country** | United States of America | |
| **Characteristics and external validity – is this study and its results generalizable to my systematic review question?** | | |
| **Patient/population/ participants** | Women with untreated PCOS were recruited from the Division of Women’s Health at the Brigham and Women’s Hospital and the Reproductive Endocrine Unit of the Massachusetts General Hospital. All women were overweight (BMI > 28 kg/m^2^), healthy and between the ages of 18 and 45 years. They were not currently taking any medications. No woman had an elevated plasma PRL level. Women in both groups were without evidence of other diseases including diabetes and hypertension. | |
| **Control population** | Age- and weight-matched control women were recruited by means of advertisement within the community and had normal menstrual cycles (28–35 days), no clinical signs of androgen excess, and normal serum levels of androgens. | |
| **PCOS diagnostic criteria** | NIH 1992; Chronic oligomenorrhea (six or fewer menses per year) along with elevated serum androgen levels (total or biologically available testosterone levels). Nonclassical 21-hydroxylase deficiency was excluded by a 1-h ACTH stimulation test. | |
| **N per group** | PCOS group – 18  Non-PCOS group – 18 | |
| **Setting** | Hospital for cases, community based recruitment through adverts for controls. | |
| **Outcomes (primary and other) with definition/tool (eg. self-reported, fasting etc.)** | Primary outcomes: Polysomnography records classified according to American Academy  of Sleep Medicine  ‐ AHI >5 [n (%)]  ‐ AHI >10 [n (%)]  ‐ AHI >15 [n (%)]  Outcomes not relevant:  ‐ Epworth Sleepiness Score (ESS questionnaire) [mean (SEM)]  ‐ Sleep onset latency [mean (SEM)]  ‐ Sleep efficiency (%) [mean (SEM)]  ‐ REM sleep (%) [mean (SEM)]  ‐ AHI (all sleep stages) [mean (SEM)]  ‐ AHI (REM sleep) [mean (SEM)] | |
| **Does the study have a clearly focused question and/or PICO?** | Yes  Partial  No  Not reported | Yes |
| **Inclusion criteria** | Yes  Partial  No  Not reported | Partial  Overweight women (Body Mass Index > 28 kg/m2). Between the ages of 18 and 45 years. For controls - normal menstrual cycles (28–35 days), and normal serum levels of androgens. Generalisable to overweight BMI ≥28 PCOS women on no medication and no other disease. |
| **Exclusion criteria** | Yes  Partial  No  Not reported | Yes  Not currently taking medication. No other disease. No elevated PRL. No nonclassical 21-hydroxylase deficiency. For controls - no clinical signs of androgen excess. |
| **If there were specified inclusion/exclusion criteria, were these appropriate?** | Yes  Partial  No  Not reported | Yes |
| **Is a cross sectional or case-control study the appropriate design to answer this question?** | Yes  Partial  No  Not reported | Yes  This study is cross-sectional. |
| **Was there sufficient duration of follow-up for outcomes to occur?** | Yes  Partial  No  Not reported | Not relevant (the study is cross-sectional in nature). |
| **Was matching performed?** | Yes  Partial  No  Not reported | Yes  Age and weight. |
| Summary Result/s | Obese women with PCOS are at increased risk of OSA when compared with matched reproductively normal women. | |
| **Internal validity – has this study been conducted rigorously in order to reduce bias?** | | |
| **Selection bias** | | |
| **Were the cases and controls taken from comparable populations?** | Yes  Partial  No  Not reported | No  Control women were recruited by means of advertisement within the community.  Women with untreated PCOS were recruited from the Division of Women’s Health at the Brigham and Women’s Hospital and the Reproductive Endocrine Unit of the Massachusetts General Hospital. |
| **Was the case definition adequate and established in a standard, valid, and**  **reliable way?** | Yes  Partial  No  Not reported | Yes  Case definition – (NIH criteria) - Chronic oligomenorrhea (six or fewer menses per year) along with elevated serum androgen levels (total or biologically available testosterone levels). A single fasting blood sample for hormone analysis was obtained  between 0800 and 1000 h. Assays for serum Testosterone (T) and DHEAS were performed by using diagnostic Products (Los Angeles, CA) Coat-A-Count kits. |
| **Was the control status established in a standard, valid and reliable way?** | Yes  Partial  No  Not reported | Yes  Control definition - Control women had normal menstrual cycles (28–35 days), no clinical signs of androgen excess, and normal serum levels of androgens. A single fasting blood sample for hormone analysis was obtained between 0800 and 1000 h. Assays for serum Testosterone (T) and DHEAS were performed by using diagnostic Products (Los Angeles, CA) Coat-A-Count kits. |
| **Performance bias** | | |
| **Aside from the exposure/intervention, were the groups treated the same?** | Yes  Partial  No  Not reported | Yes  Both groups were recruited consecutively, and, to avoid any potential recruitment bias no questions regarding symptoms of any sleep disorder were asked. |
| **Detection bias** | | |
| **Were measurements (for exposures or outcomes) carried out and calculated in**  **a standard, valid and reliable way?** | Yes  Partial  No  Not reported | Yes  A single fasting blood sample for hormone analysis was obtained between 0800 and 1000 h. Assays for serum Testosterone (T) and DHEAS were performed by using diagnostic Products (Los Angeles, CA) Coat-A-Count kits. Polysomnography was performed according to standard laboratory protocol. Data recorded included four channels of EEG (two central and two occipital), two channels of EOG, submental EMG, arterial oxygen saturation (Healthdyne, Model 930, Marietta, GA.), nasaloral airflow (thermistor), nasal pressure (Validyne Engineering Corp., Northridge CA), EKG, chest and abdominal wall motion (piezo electrodes, Pro-Tech Services, Woodinville, WA) bilateral anterior tibialis EMG, snoring (tracheal microphone) and body position (mercury gauge). All signals were simultaneously recorded and stored using the ALICE 3 digital polysomnography system (Respironics, Inc., Murraysville, PA). Bedtime was set between 2200 and 2300 h, and waketime occurred between 0600 and 0700 h. |
| **Were outcome assessors blind to case and control status?** | Yes  Partial  No  Not reported | Yes  All of the polysomnographic records were scored by one of the authors (SDP) who was blinded to all subjects’ diagnosis. |
| **Were all outcomes measured in a standard, valid and reliable way?** | Yes  Partial  No  Not reported | Yes  Polysomnography was performed according to standard laboratory protocol. Data recorded included four channels of EEG (two central and two occipital), two channels of EOG, submental EMG, arterial oxygen saturation (Healthdyne, Model 930, Marietta, GA.), nasaloral airflow (thermistor), nasal pressure (Validyne Engineering  Corp., Northridge CA), EKG, chest and abdominal wall motion (piezo electrodes, Pro-Tech Services, Woodinville, WA) bilateral anterior tibialis EMG, snoring (tracheal microphone) and body position (mercury gauge). All signals were simultaneously recorded and stored using the ALICE 3 digital polysomnography system (Respironics, Inc., Murraysville, PA). Bedtime was set between 2200 and 2300 h, and waketime occurred between 0600 and 0700 h. |
| **Were outcomes assessed objectively and**  **independently?** | Yes  Partial  No  Not reported | Yes  All of the polysomnographic records were scored by one of the authors (SDP) who was blinded to all subjects’ diagnosis. |
| **Attrition bias** | | |
| **What percentage of the individuals recruited into each arm of the study were lost to follow up?** | Yes  Partial  No  Not reported | Not relevant to cross‐sectional study. |
| **What percentage of the individuals were not included in the analysis?** | Yes  Partial  No  Not reported | 0%  All those who completed the sleep study were included in the study, regardless of the results. |
| **Report bias** | | |
| **Is the paper free of selective outcome reporting?** | Yes  Partial  No  Not reported | Not reported  No protocol or PROSPERO. |
| **Confounding** | | |
| **Are the cohorts comparable on the basis of design or analysis?** | Yes  Partial  No  Not reported | Yes  Limited baseline demographic variables reported (age, BMI, waist hip ratio, testosterone, non-SHBG bound testosterone) PCOS and control women were well matched for age and BMI. However, as can be seen, women with PCOS had a significantly higher waist-hip ratio (WHR) than control women (0.88 ± 0.02 vs.  0.82 ± 0.01, P <0.001). Women with PCOS also had significantly higher circulating testosterone (94.44 ± 8.5 ng/dl vs. 22.77 ± 2.5 ng/dl, P < 0.001) and unbound testosterone levels (34.06 ± 2.3 ng/dl vs. 5.05 ± 0.8 ng/dl P < 0.001) than controls. |
| **Other bias** | | |
| **Were there any conflicts of interest in the writing or funding of this study?** | Yes  Partial  No  Not reported | Not reported |
| **Was the study sufficiently powered to detect any differences between the groups?** | Yes  Partial  No  Not reported | Yes  First, the sample size is relatively small. However, the group differences were sufficiently robust that a larger sample size was not required to demonstrate substantial and significant differences. |
| **If statistical analysis was undertaken, was this appropriate?** | Yes  Partial  No  Not reported | Not reported  Only descriptive statistics reported. |
| **Comments** | Possible selection bias: Case and control group were recruited differently.  Did not report absolute number for OSA, only reported %. | |
| **What is the overall risk of bias?** | Low  Moderate  High Insufficient  information | Moderate |
| **Did risk of bias differ by outcome (eg.**  **primary outcome was low risk but rest**  **were high)?** | No | |

| **Study ID** | Hachul et al. 2019 | |
| --- | --- | --- |
| **Study Citation** | Hachul H, Polesel DN, Tock L, Carneiro G, Pereira AZ, Zanella MT, Tufik S, Togeiro SM.  Sleep disorders in polycystic ovary syndrome: influence of obesity and hyperandrogenism.  Rev Assoc Med Bras (1992). 2019 Mar;65(3):375-383. doi: 10.1590/1806-9282.65.3.375 | |
| **Study country** | Brazil | |
| **Characteristics and external validity – is this study and its results generalizable to my systematic review question?** | | |
| **Patient/population/ participants** | A total of 44 subjects were selected to participate in the study. The volunteers, ranging in age from 16 to 45 years, were recruited from the Endocrinology Division of the Federal University of São Paulo, Brazil. | |
| **Control population** | The control group was comprised of 14 women (17 women were originally eligible, of  which 3 excluded because of missing data on PSQI and BMI). Inclusion criteria: a regular menstrual cycle of 28-30 days, normal BMI and in the follicular phase of the menstrual cycle. Exclusion criteria: neurologic conditions and/or being under psychiatric treatment; use of medication for chronic diseases that might interfere with the study results; participation in another clinical study or having participated in a clinical study within a period of 3 months; being a carrier of a disease; having a history of stroke; use of hypnotic, psychotropic, psychostimulant, and/or analgesic drugs; use of hormonal contraceptives; and presence of dysmenorrhea or endometriosis that may interfere with sleep patterns. | |
| **PCOS diagnostic criteria** | The diagnosis of PCOS was based on the latest 2003 Rotterdam consensus, requiring the  presence of at least two of the following features: (1) oligomenorrhea or chronic anovulation,  (2) clinical and/or biochemical hyperandrogenism, and (3) ultrasound appearance of polycystic ovaries. | |
| **N per group** | PCOS were classified according to presence and absence of hyperandrogenism (Total n =30)  ‐ With hyperandrogenism (n=14)  ‐ Without hyperandrogenism (n=16)  Non-PCOS group (n=14) | |
| **Setting** | Hospital | |
| **Outcomes (primary and other) with definition/tool (eg. self-reported, fasting etc.)** | Primary outcomes:  ‐ Meet criteria for OSA: (AHI≥5+ sleep complaints) or AHI≥15 (full night polysomnography)  [n (%)]  Outcomes not relevant:  ‐ High risk of OSA (Berlin Questionnaire) [n (%)]  ‐ High daytime sleepiness (Epworth Sleepiness Scale, score≥10) [n (%)]  ‐ Poor sleep quality (PSQI, score>5) [n (%)]  ‐ Reported snoring [n (%)]  ‐ Epworth Sleepiness Score (ESS Questionnaire) [Mean (SD)]  ‐ Pittsburgh Sleep Quality Index score [Mean (SD)]  ‐ Sleep latency (min) [Mean (SD)]  ‐ REM latency (min) [Mean (SD)]  ‐ Total sleep time (min) [Mean (SD)]  ‐ Sleep efficiency (%) [Mean (SD)]  ‐ N1 sleep stage (% TST) [Men (SD)]  ‐ N2 sleep stage (% TST) [Mean (SD)]  ‐ N3 sleep stage (% TST) [Mean (SD)]  ‐ REM sleep stage (% TST) [Mean (SD)]  ‐ Wake After Sleep Onset (WASO) (min) [Mean (SD)]  ‐ Arousal index (events/h) [Mean (SD)]  ‐ Apnoea Hypopnoea Index (AHI) (events/h) [Mean (SD)]  ‐ Basal Oxygen Saturation [Mean (SD)]  ‐ Mean Oxygen Saturation [Mean (SD)]  ‐ Minimum Oxygen Saturation [Mean (SD)] | |
| **Does the study have a clearly focused question and/or PICO?** | Yes  Partial  No  Not reported | Yes |
| **Inclusion criteria** | Yes  Partial  No  Not reported | Yes  The volunteers, ranging in age from 16 to 45 years, were recruited from the Endocrinology Division of the Federal University of São Paulo, Brazil. |
| **Exclusion criteria** | Yes  Partial  No  Not reported | Yes  Subjects with other known causes of hyperandrogenism (such as congenital adrenal hyperplasia, androgen-secreting tumors and Cushing’s syndrome), using oral contraceptives, corticosteroids, antidiabetic or lipid-lowering drugs in the previous 3 months, having a history of liver disease (such as viral hepatitis B and C,  hemochromatosis and autoimmune hepatitis), diabetes mellitus, untreated hypothyroidism, renal, hepatic, cardiac or pulmonary disease, receiving treatment  for sleep apnea using medications that alter liver enzymes, with a daily ingestion of more than 20 grams of ethanol, using drugs (sympathomimetics, sympatholytics, and β-blockers), with depression or with chronic diseases were excluded. |
| **If there were specified inclusion/exclusion criteria, were these appropriate?** | Yes  Partial  No  Not reported | Partial  Partial generalizability due to exclusion of patients using oral contraceptives, and other concurrent comorbidity diagnoses that commonly co-exist among women with PCOS. |
| **Is a cross sectional or case-control study the appropriate design to answer this question?** | Yes  Partial  No  Not reported | Yes  This study is cross-sectional. |
| **Was there sufficient duration of follow-up for outcomes to occur?** | Yes  Partial  No  Not reported | Not relevant (the study is cross-sectional in nature). |
| **Was matching performed?** | Yes  Partial  No  Not reported | No |
| Summary Result/s | Only the PCOS group had obstructive sleep apnoea diagnosis in this study. | |
| **Internal validity – has this study been conducted rigorously in order to reduce bias?** | | |
| **Selection bias** | | |
| **Were the cases and controls taken from comparable populations?** | Yes  Partial  No  Not reported | Yes  The volunteers (exposed and unexposed), ranging in age from 16 to 45 years, were recruited from the Endocrinology Division of the Federal University of São Paulo, Brazil. |
| **Was the case definition adequate and established in a standard, valid, and**  **reliable way?** | Yes  Partial  No  Not reported | Yes  The diagnosis of PCOS was based on the latest 2003 Rotterdam consensus,18 requiring the presence of at least two of the following features: (1) oligomenorrhea or chronic anovulation, (2) clinical and/or biochemical hyperandrogenism, and (3) ultrasound appearance of polycystic ovaries. |
| **Was the control status established in a standard, valid and reliable way?** | Yes  Partial  No  Not reported | Yes  As above. |
| **Performance bias** | | |
| **Aside from the exposure/intervention, were the groups treated the same?** | Yes  Partial  No  Not reported | Yes |
| **Detection bias** | | |
| **Were measurements (for exposures or outcomes) carried out and calculated in**  **a standard, valid and reliable way?** | Yes  Partial  No  Not reported | Yes  Questionnaires were used to document clinical history, including regularity and length of menstrual cycles, and ovulation status. Signs of androgen excess (hirsutism, alopecia, acne) were noted in the physical examination. Hirsutism with a Ferriman-Gallwey score of 8 or above was considered clinical evidence of androgen excess.  All subjects underwent an ultrasound examination of the pelvis by the same radiologist. LOGIQ P5 (GE Healthcare®, Wauwatosa, WI) with an 8 MHz transvaginal transducer was used for the ultrasound of the pelvis. |
| **Were outcome assessors blind to case and control status?** | Yes  Partial  No  Not reported | Not reported |
| **Were all outcomes measured in a standard, valid and reliable way?** | Yes  Partial  No  Not reported | Partial  Full-night polysomnography (PSG) was performed, using a digitalsystem (EMBLA®  S700®, Embla Systems Inc, Broomfield, CO) at the sleep laboratory for one night. Trained technicians visually scored all of the PSG data according to standardized criteria for investigating sleep. Electroencephalogram arousals and sleep-related  respiratory events were scored following the criteria outlined in the American Academy of Sleep Medicine Manual for Scoring Sleep and Associated Events. OSA classification was defined according to the AHI. Participants were diagnosed with OSA if they presented an AHI≥5 and sleep complaints. Participants with an AHI≥15 were diagnosed with OSA, regardless of whether they had any additional complaint.  Unclear what was considered as a sleep complaint. |
| **Were outcomes assessed objectively and**  **independently?** | Yes  Partial  No  Not reported | Partial  No report of independent outcome assessment, i.e., blinding outcome assessors to patient’s exposure status. However, trained technicians visually scored all of the PSG data according to standardized criteria for investigating sleep. |
| **Attrition bias** | | |
| **What percentage of the individuals recruited into each arm of the study were lost to follow up?** | Yes  Partial  No  Not reported | Not relevant to cross‐sectional study. |
| **What percentage of the individuals were not included in the analysis?** | Yes  Partial  No  Not reported | 20% overall. From a total of 55 women initially included in the study, 11 individuals were excluded because of missing data (8 related to the PSQI and 3 to BMI). |
| **Report bias** | | |
| **Is the paper free of selective outcome reporting?** | Yes  Partial  No  Not reported | Yes  The study was approved by the Ethics Committee for Research of the Federal University of Sao Paulo (#0588/2010). |
| **Confounding** | | |
| **Are the cohorts comparable on the basis of design or analysis?** | Yes  Partial  No  Not reported | Yes  The authors observed a higher BMI in the PCOS group (F1,42=36,404; P<0.001) compared to the control group. |
| **Other bias** | | |
| **Were there any conflicts of interest in the writing or funding of this study?** | Yes  Partial  No  Not reported | No |
| **Was the study sufficiently powered to detect any differences between the groups?** | Yes  Partial  No  Not reported | Not reported |
| **If statistical analysis was undertaken, was this appropriate?** | Yes  Partial  No  Not reported | Yes  Pearson’s chi-squared test was performed to determine the association between categorical variables. BMI and age were used as adjustment factors in evaluating the effect of PCOS and hyperandrogenism on sleep, respectively. The results were submitted to adjustment only when the groups had significant statistical differences in age or BMI. |
| **Comments** | Selection bias due to 20% patients excluded from the analysis.  Outcome assessment was not completely objective, and no report of blinding the outcome assessors to patient’s exposure status. | |
| **What is the overall risk of bias?** | Low  Moderate  High Insufficient  information | Moderate |
| **Did risk of bias differ by outcome (eg.**  **primary outcome was low risk but rest**  **were high)?** | No | |

| **Study ID** | Nandalike et al. 2012 | |
| --- | --- | --- |
| **Study Citation** | Nandalike K, Agarwal C, Strauss T, Coupey SM, Isasi CR, Sin S, Arens R. Sleep and  cardiometabolic function in obese adolescent girls with polycystic ovary syndrome. Sleep  Med. 2012 Dec;13(10):1307-12. doi: 10.1016/j.sleep.2012.07.002 | |
| **Study country** | United States of America | |
| **Characteristics and external validity – is this study and its results generalizable to my systematic review question?** | | |
| **Patient/population/ participants** | 28 adolescent girls aged 13–18 years diagnosed with PCOS and followed at Children’s Hospital at Montefiore (CHAM), between January 2006 and December 2009, who were subsequently referred for an overnight polysomnography (PSG) at CHAM to rule out OSA, because of sleep-related complaints such as snoring, trouble breathing or excessive daytime sleepiness. Referral for PSG was through the adolescent medicine, endocrine, otolaryngology and pulmonary clinics for sleep-related complaints such as snoring, trouble breathing at night or excessive daytime sleepiness. Participants were first identified by an electronic medical information database (Clinical Looking Glass, CLG). | |
| **Control population** | Age- and body mass index (BMI) Z-score-matched females without PCOS (n=28) and BMI Z-score matched males (n=28) who underwent PSG during the same time period were identified through the sleep–disorders centre database (referred to sleep disorder centre for sleep related complaints).  Charts of females chosen as controls were verified and any girl with any history of oligomenorrhea (less than nine menstrual cycles in a year) or amenorrhoea, or any documented clinical signs of hyperandrogenism such as acne or hirsutism or biochemical evidence of hyperandrogenemia, was excluded from the study. | |
| **PCOS diagnostic criteria** | Diagnosis of PCOS was made as per the modified Rotterdam criteria. Accordingly, at least  two of the following three features existed: (1) oligomenorrhea/amenorrhea, (2) clinical or biochemical evidence of hyperandrogenemia and (3) polycystic ovaries documented on ultrasonography. In our  sample, all of the patients fulfilled the first two criteria and only a few had ultrasonography  performed, as the interpretation of the sonographic findings is different for adolescents who  may have multicystic ovaries as a normal peripubertal finding. Other conditions that could  mimic PCOS such as Cushing’s syndrome, late onset adrenal hyperplasia or androgen producing neoplasm were excluded. | |
| **N per group** | Women with PCOS (n=28)  Control women (n=28)  Control men (n=28) | |
| **Setting** | Hospital | |
| **Outcomes (primary and other) with definition/tool (eg. self-reported, fasting etc.)** | Primary outcomes:  ‐ OSA: OSA was diagnosed if the apnoea hypopnoea index (AHI)  was more than 5/h or if the apnoea index was more than 1/h [n (%)]  Outcomes not relevant:  ‐ Sleep onset latency (minutes) [Mean (SD)]  ‐ Sleep efficiency (%) [Mean (SD)]  ‐ Arousal awakening index (events/h) [Mean (SD)]  ‐ AHI (events/h) [Mean (SD)]  ‐ Baseline oxygen (%) [Mean (SD)]  ‐ Lowest oxygen (%) [Mean (SD)]  ‐ Peak ETCO2 (mmHg) [Mean (SD)]  ‐ MetS (n) (%) [Mean (SD)]  ‐ Fasting glucose (mg/dL) [Mean (SD)]  ‐ Fasting insulin (IU) [Mean (SD)]  ‐ HOMA-IR>4 [n (%)]  ‐ Fasting TG (mg/dL) [Mean (SD)]  ‐ Fasting HDL (mg/dL) [Mean (SD)]  ‐ Systolic blood pressure (mmHg) [Mean (SD)]  ‐ Diastolic blood pressure (mmHg) [Mean (SD)] | |
| **Does the study have a clearly focused question and/or PICO?** | Yes  Partial  No  Not reported | Yes |
| **Inclusion criteria** | Yes  Partial  No  Not reported | Partial  Limited to adolescents aged 13–18 years, and only those who were referred for a polysomnography to rule out OSA as they had sleep related complaints (so biased towards those who already had possible symptoms of OSA). |
| **Exclusion criteria** | Yes  Partial  No  Not reported | Yes  Patients with significant co-morbid conditions contributing to OSA, such as Trisomy 21, craniofacial anomalies and cerebral palsy were also excluded from the study. |
| **If there were specified inclusion/exclusion criteria, were these appropriate?** | Yes  Partial  No  Not reported | Yes |
| **Is a cross sectional or case-control study the appropriate design to answer this question?** | Yes  Partial  No  Not reported | Yes  This study is cross-sectional. |
| **Was there sufficient duration of follow-up for outcomes to occur?** | Yes  Partial  No  Not reported | Not relevant (the study is cross-sectional in nature). |
| **Was matching performed?** | Yes  Partial  No  Not reported | Yes, age- and body mass index (BMI) Z-score-matched. |
| Summary Result/s | The authors report a higher prevalence of OSA and metabolic dysfunction in a selected group of obese girls with PCOS referred with sleep-related complaints compared to BMI-matched control girls without PCOS. | |
| **Internal validity – has this study been conducted rigorously in order to reduce bias?** | | |
| **Selection bias** | | |
| **Were the cases and controls taken from comparable populations?** | Yes  Partial  No  Not reported | Partial  Exposed patients were first identified by an electronic medical information database (Clinical Looking Glass, CLG) who were referred to the sleep-disorders centre for PSG. Unexposed patients were directly identified through the sleep –disorders centre  database. |
| **Was the case definition adequate and established in a standard, valid, and**  **reliable way?** | Yes  Partial  No  Not reported | Yes    Participants were first identified by an electronic medical information database (Clinical Looking Glass, CLG). Accordingly, the PCOS ICD-9 code-256.4 was queried, and the diagnosis was verified by reviewing each patient’s electronic patient file (EPF). Later, individual charts were reviewed to identify the individuals who underwent PSG during the study period. |
| **Was the control status established in a standard, valid and reliable way?** | Yes  Partial  No  Not reported | Partial  Charts of females chosen as controls were verified and any girl with any history of oligomenorrhea (less than nine menstrual cycles in a year) or amenorrhoea, or any documented clinical signs of hyperandrogenism such as acne or hirsutism or biochemical evidence of hyperandrogenemia, was excluded from the study. The androgen profile was not available for any of the female controls as they had no menstrual irregularities or clinical hyperandrogenism and were not biochemically tested for excess androgen. |
| **Performance bias** | | |
| **Aside from the exposure/intervention, were the groups treated the same?** | Yes  Partial  No  Not reported | No  This is a retrospective chart review of routine health records, so we cannot be sure the groups were treated the same. |
| **Detection bias** | | |
| **Were measurements (for exposures or outcomes) carried out and calculated in**  **a standard, valid and reliable way?** | Yes  Partial  No  Not reported | Partial  Conducted as part of routine care. The data on total and free-serum testosterone level were collected on all the subjects in whom it was available. Total testosterone level was available on all girls with PCOS and free testosterone level was available on 25/28 girls with PCOS. The androgen profile was not available for any of the female controls as they had no menstrual irregularities or clinical hyperandrogenism and were not biochemically tested for excess androgens. |
| **Were outcome assessors blind to case and control status?** | Yes  Partial  No  Not reported | Not reported |
| **Were all outcomes measured in a standard, valid and reliable way?** | Yes  Partial  No  Not reported | Partial  Conducted as part of routine care. PSG data (via Xltek, Oakville, ON, Canada) were extracted from the electronic records of the sleep-disorders centre at CHAM. Only  28/240 girls with PCOS were referred by their primary care physicians for a PSG to evaluate for OSAS. Information on any sleep-related complaints or any screening measures for OSA on the 212 girls with PCOS not referred for PSG was unavailable. |
| **Were outcomes assessed objectively and**  **independently?** | Yes  Partial  No  Not reported | Yes  Sleep staging and scoring of arousals were performed as per standard criteria. OSA was diagnosed if the apnoea hypopnoea index (AHI) was more than 5/h or if the apnoea index was more than 1/h. |
| **Attrition bias** | | |
| **What percentage of the individuals recruited into each arm of the study were lost to follow up?** | Yes  Partial  No  Not reported | Not relevant to cross‐sectional study. |
| **What percentage of the individuals were not included in the analysis?** | Yes  Partial  No  Not reported | 88.33% of the girls with PCOS. Only 28/240 girls with PCOS were referred by their primary care physicians for a PSG to evaluate for OSAS. |
| **Report bias** | | |
| **Is the paper free of selective outcome reporting?** | Yes  Partial  No  Not reported | Not reported  No protocol or PROSPERO |
| **Confounding** | | |
| **Are the cohorts comparable on the basis of design or analysis?** | Yes  Partial  No  Not reported | No  Compared to the study group girls with PCOS, the female control group had a higher proportion of African Americans (5/28(17.9%) vs. 13/28(46.4%). Also, a greater proportion of girls from the PCOS group were prescribed metformin compared to the female control groups (10/28 (35.7%) vs. 3/28 (10.7%). Similarly, a higher proportion of girls from the PCOS group had a history of adenotonsillectomy prior to PSG  compared to the female control groups (9/28 (32.1%) vs. 3/28 (10.7%)). |
| **Other bias** | | |
| **Were there any conflicts of interest in the writing or funding of this study?** | Yes  Partial  No  Not reported | No |
| **Was the study sufficiently powered to detect any differences between the groups?** | Yes  Partial  No  Not reported | Not reported |
| **If statistical analysis was undertaken, was this appropriate?** | Yes  Partial  No  Not reported | Yes  Mean and standard deviation were used to summarise continuous variables. Analysis of variance (ANOVA) was used to compare PSG findings, cardiometabolic profiles and other continuous variables between the PCOS subjects and control groups.  Differences in proportions were assessed with the Chi-square test. |
| **Comments** | Only women who were referred for PSG were included.  Highly susceptible to referral bias. | |
| **What is the overall risk of bias?** | Low  Moderate  High Insufficient  information | High |
| **Did risk of bias differ by outcome (eg.**  **primary outcome was low risk but rest**  **were high)?** | No | |

| **Study ID** | Suri et al. 2016 | |
| --- | --- | --- |
| **Study Citation** | Suri J, Suri JC, Chatterjee B, Mittal P, Adhikari T. Obesity may be the common pathway for  sleep-disordered breathing in women with polycystic ovary syndrome. Sleep Med. 2016  Aug;24:32-39. doi: 10.1016/j.sleep.2016.02.014 | |
| **Study country** | India | |
| **Characteristics and external validity – is this study and its results generalizable to my systematic review question?** | | |
| **Patient/population/ participants** | 50 patients with PCOS who attended the Gynecology Outpatient Department (OPD) and Reproductive Endocrinology Clinic of Vardhman Mahavir Medical College and Safdarjung Hospital, New Delhi, India who met inclusion and exclusion criteria and consented to take part in the study. | |
| **Control population** | A total of 100 age-matched women who attended the gynecology OPD with other complaints such as vaginal discharge, dysuria, and pelvic organ prolapse were recruited as control subjects after obtaining the required consent. All of these women experienced regular menstrual cycles and did not meet the standard diagnostic criteria for PCOS. | |
| **PCOS diagnostic criteria** | PCOS was defined by the Rotterdam criteria, namely, the presence of any two of the following three features: (1) chronic oligomenorrhea (six or fewer spontaneous menses per year), (2) biochemical or clinical evidence of hyperandrogenism, and (3) polycystic ovaries on ultrasonography. | |
| **N per group** | Women with PCOS (n=50)  Control women (n=100) | |
| **Setting** | Hospital | |
| **Outcomes (primary and other) with definition/tool (eg. self-reported, fasting etc.)** | Primary outcomes:  ‐ Sleep disordered breathing (Polysomnography)  SDB was defined as an RDI≥ along with symptoms such as EDS, choking, witnessed  apneic spell, nocturia, or an RDI>15 with or without associated symptoms.  Outcomes not relevant:  ‐ Snoring (patient reported)  ‐ Respiratory Distress Index (RDI) [Mean (SD)]  ‐ Snoring (PSG document) [n (%)]  ‐ Sleep onset [Mean (SD)]  ‐ Total Sleep Time [Mean (SD)]  ‐ Wake After Sleep Onset (WASO) [Mean (SD)]  ‐ Rapid Eye Movement (REM) [Mean (SD)]  ‐ Non-Rapid Eye Movement (NREM) [Mean (SD)]  ‐ Sleep Efficiency (SE) (%) [Mean (SD)]  ‐ Epworth Sleepiness Scale (ESS) [Mean (SD) | |
| **Does the study have a clearly focused question and/or PICO?** | Yes  Partial  No  Not reported | Yes |
| **Inclusion criteria** | Yes  Partial  No  Not reported | Partial  Mention of inclusion criteria, but not clearly reported. Those who gave consent to take part in the study. |
| **Exclusion criteria** | Yes  Partial  No  Not reported | Yes  Women taking any form of treatment for PCOS were not included in the study. Patients with thyroid disorders, hyperprolactinemia, and congenital adrenal hyperplasia, with history of smoking, and with neurological or psychiatric disorders were also excluded from the study. |
| **If there were specified inclusion/exclusion criteria, were these appropriate?** | Yes  Partial  No  Not reported | Partial  Unclear why women taking treatment for PCOS and with comorbidities were excluded from the study. |
| **Is a cross sectional or case-control study the appropriate design to answer this question?** | Yes  Partial  No  Not reported | Yes  This study is cross-sectional. |
| **Was there sufficient duration of follow-up for outcomes to occur?** | Yes  Partial  No  Not reported | Not relevant (the study is cross-sectional in nature). |
| **Was matching performed?** | Yes  Partial  No  Not reported | Yes  Age |
| Summary Result/s | SDB was seen in 66% of the case patients and in 4% of control group with (odds ratio [OR]  = 46.5, 95% confidence interval [CI] = 14.6–148.4; p < 0.001). After adjustment for body  mass index (BMI) and waist circumference (WC), the difference was not significant (p =  0.993 and p = 0.931, respectively). | |
| **Internal validity – has this study been conducted rigorously in order to reduce bias?** | | |
| **Selection bias** | | |
| **Were the cases and controls taken from comparable populations?** | Yes  Partial  No  Not reported | Yes  Both cases and controls attended the Gynecology Outpatient Department (OPD). |
| **Was the case definition adequate and established in a standard, valid, and**  **reliable way?** | Yes  Partial  No  Not reported | Yes  PCOS was defined by the Rotterdam criteria, namely, the presence of any two of the following three features: (1) chronic oligomenorrhea (six or fewer spontaneous menses per year), (2) biochemical or clinical evidence of hyperandrogenism, and (3) polycystic ovaries on ultrasonography. |
| **Was the control status established in a standard, valid and reliable way?** | Yes  Partial  No  Not reported | Yes  Control women experienced regular menstrual cycles and did not meet the standard diagnostic criteria for PCOS. All women underwent clinical evaluation, physical examination, and hormonal tests (including testosterone and DHEAS). |
| **Performance bias** | | |
| **Aside from the exposure/intervention, were the groups treated the same?** | Yes  Partial  No  Not reported | No  Keeping in mind the cost and difficulty of convincing a normal asymptomatic woman to undergo a sleep study, we did not find it practical to subject all of the controls to the overnight PSG. Instead, only those women who reported snoring underwent the overnight PSG, as snoring was considered to be a surrogate marker for SDB. Not all control participants had polysomnography, only 16 / 100 who reported snoring had it done due to costs and difficulty consenting. |
| **Detection bias** | | |
| **Were measurements (for exposures or outcomes) carried out and calculated in**  **a standard, valid and reliable way?** | Yes  Partial  No  Not reported | Partial  Overnight in-laboratory polysomnography (PSG), the hormonal and biochemical assays of all case patients and control subjects were done on the second or third day of the menstrual cycle. PSG was done in all 50 women with PCOS, whereas in the control group, it was done only in the 16 women who reported snoring. PSG data that were recorded included a three-channel electroencephalography (EEG), two-channel  electrooculography, anterior tibialis and submental electromyography, nasal airflow by thermistor, nasal pressure by pressure cannula, thoracic and abdominal efforts by strain gauges, oxygen saturation by pulse oximeter, and tracheal sound with microphone attached to the neck. The EEG channels used were F3M2, C3M2, and O1M2. ALICE 5 digital polysomnography system (Respironics, Murrysville, PA) was used for recording and storing all of the signals. A length of at least seven hours of sleep was recorded in each subject. |
| **Were outcome assessors blind to case and control status?** | Yes  Partial  No  Not reported | Not reported |
| **Were all outcomes measured in a standard, valid and reliable way?** | Yes  Partial  No  Not reported | No  PSG was done in all 50 women with PCOS, whereas in the control group, it was done only in the 16 women who reported snoring. Apnea was defined as a drop in the peak thermal sensor excursion by more than 90% of the baseline lasting for ten seconds or more. Hypopnea was the drop of nasal pressure signal excursions by more than 30% of the baseline lasting for more than ten seconds and was accompanied by a drop in 3% or more of oxygen saturation from the pre-event baseline or an arousal. Respiratory effort–related arousal (RERA) was termed as an event of increasing the respiratory effort or flattening nasal pressure waveform for more than ten seconds, followed by an arousal from sleep, which does not meet the criteria for apnea or hypopnea. Respiratory distress Index (RDI) was defined as the number of obstructive apneas, hypopneas, and RERAs per hour of sleep. This was calculated by dividing the total  number of respiratory events by the total sleep time in hours. |
| **Were outcomes assessed objectively and**  **independently?** | Yes  Partial  No  Not reported | Yes  SDB was defined as an RDI of five or more along with symptoms such as EDS, Choking, witnessed apneic spell, nocturia, or an RDI >15 with or without associated symptoms. The severity of SDB was defined according to the RDI as mild (5–15/h), moderate (16–30/h), and severe (>30/h). Polysomnography all scored by an experienced sleep medicine consultant. |
| **Attrition bias** | | |
| **What percentage of the individuals recruited into each arm of the study were lost to follow up?** | Yes  Partial  No  Not reported | Not relevant to cross‐sectional study |
| **What percentage of the individuals were not included in the analysis?** | Yes  Partial  No  Not reported | 16.66% in the treatment group.  Of the 60 patients who fulfilled the criteria, 50 gave their consent to take part in the study. |
| **Report bias** | | |
| **Is the paper free of selective outcome reporting?** | Yes  Partial  No  Not reported | Not reported  No protocol or PROSPERO |
| **Confounding** | | |
| **Are the cohorts comparable on the basis of design or analysis?** | Yes  Partial  No  Not reported | Yes  The mean age of the two groups was comparable. However, the BMI  and waist circumference were significantly higher in the case patients  when compared with the control subjects (p < 0.001; Table 1). |
| **Other bias** | | |
| **Were there any conflicts of interest in the writing or funding of this study?** | Yes  Partial  No  Not reported | No |
| **Was the study sufficiently powered to detect any differences between the groups?** | Yes  Partial  No  Not reported | Not reported |
| **If statistical analysis was undertaken, was this appropriate?** | Yes  Partial  No  Not reported | Yes  The difference in prevalence of snoring and OSA categories between patients with PCOS and control subjects was assessed using the Chi-square test (SPSS, version 19; SPSS Inc., Chicago, IL). Multivariate regression analysis was used for eliminating the confounding effect of BMI and waist circumference. |
| **Comments** | PSG was done in all 50 women with PCOS, whereas in the control group, it was done only  in the 16 women who reported snoring. | |
| **What is the overall risk of bias?** | Low  Moderate  High Insufficient  information | High |
| **Did risk of bias differ by outcome (eg.**  **primary outcome was low risk but rest**  **were high)?** | Yes  Secondary outcome not requiring PSG such as ESS have low risk of bias. | |

| **Study ID** | Tasali et al. 2008 | |
| --- | --- | --- |
| **Study Citation** | Tasali E, Van Cauter E, Hoffman L, Ehrmann DA. Impact of obstructive sleep apnea on  insulin resistance and glucose tolerance in women with polycystic ovary syndrome. J Clin  Endocrinol Metab. 2008 Oct;93(10):3878-84. doi: 10.1210/jc.2008-0925 | |
| **Study country** | United States of America | |
| **Characteristics and external validity – is this study and its results generalizable to my systematic review question?** | | |
| **Patient/population/ participants** | Women with PCOS aged between 18 and 40 years old were consecutively recruited from  the Endocrinology Clinics at the University of Chicago between February 1, 2004, and  September 30, 2007. | |
| **Control population** | During the same period of time, overweight (BMI >25 kg/m2 but <30 kg/m2) and obese (BMI ≥30 kg/m^2^) women who were otherwise healthy were recruited through public advertisements in the local community. | |
| **PCOS diagnostic criteria** | NIH 1990; A diagnosis of PCOS required 1) the presence of oligo/amenorrhea; 2) hyperandrogenemia, defined by a supranormal plasma free testosterone level (>10 pg/ml); 3) hyperandrogenism, as evidenced by infertility, hirsutism, acne, or androgenetic alopecia; and 4) exclusion of non-classic 21-hydroxylase deficiency, congenital adrenal hyperplasia, Cushing’s syndrome, hypothyroidism, or significant elevations in serum prolactin. | |
| **N per group** | Women with PCOS (n=52)  Control women (n=21) | |
| **Setting** | Hospital | |
| **Outcomes (primary and other) with definition/tool (eg. self-reported, fasting etc.)** | Primary outcomes:  ‐ OSA (AHI≥5)  ‐ Mild OSA (AHI>5 and AHI<15)  ‐ Moderate OSA (AHI>15 and AHI<30)  ‐ Severe OSA (AHI≥30) | |
| **Does the study have a clearly focused question and/or PICO?** | Yes  Partial  No  Not reported | Yes |
| **Inclusion criteria** | Yes  Partial  No  Not reported | Yes  Sleep complaints or symptoms of OSA were not used as selection criteria for the study. Only women between 18 and 40 years of age were recruited to reduce the impact of age upon ovarian function and glucose tolerance. |
| **Exclusion criteria** | Yes  Partial  No  Not reported | Yes  Subjects were excluded if they smoked cigarettes; were diabetic or hypertensive; had a history of cardiac, psychiatric, neurological, or endocrine disease; or were taking any medications at the time of the study. |
| **If there were specified inclusion/exclusion criteria, were these appropriate?** | Yes  Partial  No  Not reported | Yes |
| **Is a cross sectional or case-control study the appropriate design to answer this question?** | Yes  Partial  No  Not reported | Yes  This study is cross-sectional. |
| **Was there sufficient duration of follow-up for outcomes to occur?** | Yes  Partial  No  Not reported | Not relevant (the study is cross-sectional in nature). |
| **Was matching performed?** | Yes  Partial  No  Not reported | Yes  Age and BMI |
| Summary Result/s | Twenty-nine women (56%) with PCOS had OSA compared with four controls (19%)  (adjusted odds ratio 7.1; 95% confidence interval, 1.7–45.7; P<0.01). | |
| **Internal validity – has this study been conducted rigorously in order to reduce bias?** | | |
| **Selection bias** | | |
| **Were the cases and controls taken from comparable populations?** | Yes  Partial  No  Not reported | No  Women with PCOS aged between 18 and 40 years old were consecutively recruited from the Endocrinology Clinics at the University of Chicago between February 1, 2004, and September 30, 2007. During the same period of time, overweight (BMI >25 kg/m2 but <30 kg/m2) and obese (BMI ≥30 kg/m2) women who were otherwise healthy were recruited through public advertisements in the local community. |
| **Was the case definition adequate and established in a standard, valid, and**  **reliable way?** | Yes  Partial  No  Not reported | Yes  A complete medical history was obtained, and a physical examination was conducted in all subjects. A fasting blood sample was drawn for routine laboratory tests and the measurement of serum concentrations of total testosterone, free testosterone,  SHBG, and dehydroepiandrosterone sulfate (DHEAS). All testing was performed in the follicular phase of the menstrual cycle in normally cycling women. Progesterone levels were measured on a fasting blood sample to confirm the phase of the  menstrual cycle. |
| **Was the control status established in a standard, valid and reliable way?** | Yes  Partial  No  Not reported | Yes  As above |
| **Performance bias** | | |
| **Aside from the exposure/intervention, were the groups treated the same?** | Yes  Partial  No  Not reported | Yes |
| **Detection bias** | | |
| **Were measurements (for exposures or outcomes) carried out and calculated in**  **a standard, valid and reliable way?** | Yes  Partial  No  Not reported | Yes  A complete medical history was obtained, and a physical examination was conducted in all subjects. Overnight laboratory polysomnography was performed to establish the presence and the severity of OSA. The following morning, a fasting blood sample  was drawn for routine laboratory tests and the measurement of serum concentrations of total testosterone, free testosterone, SHBG, and dehydroepiandrosterone sulfate (DHEAS). All testing was performed in the follicular phase of the menstrual  cycle in normally cycling women. Progesterone levels were measured on a fasting blood sample to confirm the phase of the menstrual cycle. |
| **Were outcome assessors blind to case and control status?** | Yes  Partial  No  Not reported | Not reported |
| **Were all outcomes measured in a standard, valid and reliable way?** | Yes  Partial  No  Not reported | Yes  Overnight polysomnography (Neurofax EEG 1100 digital acquisition system; Nihon Kohden, Foothill Ranch, CA) included recordings of two central and two occipital electroencephalogram channels, bilateral electrooculograms, submental electromyogram, leg movements by bilateral anterior tibialis electromyogram,  electrocardiogram, oronasal airflow by thermistor, chest and abdominal wall motion by piezo electrodes, and arterial oxygen saturation by pulse oximeter. |
| **Were outcomes assessed objectively and**  **independently?** | Yes  Partial  No  Not reported | Yes  Sleep recordings were visually scored in 30-sec epochs in stages 1, 2, 3, and 4 of non-rapid eye movement sleep and in rapid eye movement sleep according to standard criteria. Obstructive respiratory events (i.e. apneas and hypopneas) and microarousals  were scored according to established criteria. The apnea-hypopnea index (AHI) was calculated as the total number of obstructive respiratory events per hour of sleep. |
| **Attrition bias** | | |
| **What percentage of the individuals recruited into each arm of the study were lost to follow up?** | Yes  Partial  No  Not reported | Not relevant to cross‐sectional study. |
| **What percentage of the individuals were not included in the analysis?** | Yes  Partial  No  Not reported | Not reported |
| **Report bias** | | |
| **Is the paper free of selective outcome reporting?** | Yes  Partial  No  Not reported | Not reported  No protocol or PROSPERO |
| **Confounding** | | |
| **Are the cohorts comparable on the basis of design or analysis?** | Yes  Partial  No  Not reported | Yes  The control group was comprised of two overweight and 19 obese women. The mean BMI among controls was 36.0±1.5 kg/m2 (range, 27.7– 48.8 kg/m2). The PCOS group  had two lean, two overweight, and 48 obese women. The mean BMI in the PCOS group was 39.2±1.0 kg/m2 (range, 23.2–58.8 kg/m2). The control group had a higher proportion of women of African-American or Hispanic descent (86 vs. 62%;  P=0.054) who have a higher risk of insulin resistance and type 2 diabetes than White women. |
| **Other bias** | | |
| **Were there any conflicts of interest in the writing or funding of this study?** | Yes  Partial  No  Not reported | No |
| **Was the study sufficiently powered to detect any differences between the groups?** | Yes  Partial  No  Not reported | Not reported |
| **If statistical analysis was undertaken, was this appropriate?** | Yes  Partial  No  Not reported | Yes  Using logistic regression, the odds ratio (with 95% confidence intervals) for having OSA in PCOS women compared with control women was calculated after adjustment for age, BMI, and ethnicity based diabetes risk (Whites, low risk; African-Americans and Hispanics, high risk). |
| **Comments** | Control women were recruited separately through public advertisements in the local community. | |
| **What is the overall risk of bias?** | Low  Moderate  High Insufficient  information | Moderate |
| **Did risk of bias differ by outcome (eg.**  **primary outcome was low risk but rest**  **were high)?** | No | |

| **Study ID** | Vgontzas et al. 2001 | |
| --- | --- | --- |
| **Study Citation** | Vgontzas AN, Legro RS, Bixler EO, Grayev A, Kales A, Chrousos GP. Polycystic ovary  syndrome is associated with obstructive sleep apnea and daytime sleepiness: role of  insulin resistance. J Clin Endocrinol Metab. 2001 Feb;86(2):517-20. doi:  10.1210/jcem.86.2.7185 | |
| **Study country** | United States of America | |
| **Characteristics and external validity – is this study and its results generalizable to my systematic review question?** | | |
| **Patient/population/ participants** | Fifty-three premenopausal women with PCOS [age range, 16–45 years; body mass index (BMI) range, 24.3–67.7] were prospectively studied in the sleep laboratory. PCOS women were recruited randomly from a larger PCOS population, and it is possible that a selection bias exists, in that those patients with sleep problems were more likely to volunteer to participate in the study. | |
| **Control population** | Control women were 452 premenopausal women 42 years of age or younger (age range, 20-42 years; BMI range, 16.1–59.9) selected from a general randomized sample. The sample for this epidemiological study was obtained using a 2-stage strategy. In the first stage of this study, a sample of women (age ≥ 20 years) was randomly selected from telephone households, and 12,219 completed a telephone interview. In the second phase of this study, a random sample from those previously interviewed by telephone was selected for study in our sleep laboratory, to assess for sleep apnea. This selection was based on risk factors reported in the telephone interview (snoring, daytime sleepiness, obesity, hypertension, and menopause), and those with a higher risk for sleep apnea were oversampled. The sleep laboratory sample consisted of 1,000 women. For analysis purposes, compensatory weights were developed to obtain estimates of prevalence of the original target population of women. | |
| **PCOS diagnostic criteria** | NIH criteria; The diagnosis of PCOS was made by the presence of chronic anovulation (six or fewer  menstrual periods per year) in association with elevated circulating androgen levels (total  testosterone more than 201.1 nmol/L and/or free and weakly bound testosterone more than 55.5 nmol/L). Non-classic adrenal 21-hydroxylase deficiency, hyperprolactinemia, and androgen secreting  tumors were excluded by appropriate tests before the diagnosis of PCOS was made. | |
| **N per group** | Women with PCOS (n=53)  Control women (n=452) | |
| **Setting** | Unclear (likely to be sleep research and treatment centre or hospital based on author affiliations) | |
| **Outcomes (primary and other) with definition/tool (eg. self-reported, fasting etc.)** | Primary outcomes:  ‐ AHI>10 per hour of sleep plus the presence of clinical symptomatology, e.g. daytime  sleepiness, hypertension, or other  cardiovascular complication (PSG + questionnaire) [n (%)]  Outcomes not relevant:  ‐ Sleep apnoea (subjective diagnosis based on requiring immediate treatment) [n (%)]  ‐ Upper airway resistance syndrome (PSG + questionnaire) [n (%)]  ‐ Sleep latency (min) [Mean (D)]  ‐ Wake Time After Sleep Onset (WTASO) (min) [Mean (SD)]  ‐ Total Wake Time (min) [Mean (SD)]  ‐ % Sleep Time [Mean (SD)]  ‐ % Slow Wave [Mean (SD)]  ‐ % Rapid Eye Movement [Mean (SD)] | |
| **Does the study have a clearly focused question and/or PICO?** | Yes  Partial  No  Not reported | Yes |
| **Inclusion criteria** | Yes  Partial  No  Not reported | Yes  Premenopausal and 42 years of age or younger. |
| **Exclusion criteria** | Yes  Partial  No  Not reported | No |
| **If there were specified inclusion/exclusion criteria, were these appropriate?** | Yes  Partial  No  Not reported | Yes |
| **Is a cross sectional or case-control study the appropriate design to answer this question?** | Yes  Partial  No  Not reported | Yes  This is a cross-sectional study. |
| **Was there sufficient duration of follow-up for outcomes to occur?** | Yes  Partial  No  Not reported | Not relevant (the study is cross-sectional in nature). |
| **Was matching performed?** | Yes  Partial  No  Not reported | No |
| Summary Result/s | OSA was much more prevalent in premenopausal women with PCOS than in normal controls (ratio, 30:1). This difference remained significant, even when we corrected for BMI differences between the two groups. | |
| **Internal validity – has this study been conducted rigorously in order to reduce bias?** | | |
| **Selection bias** | | |
| **Were the cases and controls taken from comparable populations?** | Yes  Partial  No  Not reported | No  Unclear how women with PCOS were recruited. Control women were obtained using a 2-stage strategy. |
| **Was the case definition adequate and established in a standard, valid, and**  **reliable way?** | Yes  Partial  No  Not reported | Yes  All of the PCOS women had oligo/amenorrhea and polycystic ovaries, by ultrasound examination. Seventy five percent were hirsute and nulliparous. |
| **Was the control status established in a standard, valid and reliable way?** | Yes  Partial  No  Not reported | No  Control subjects were not specifically screened for the presence of PCOS. |
| **Performance bias** | | |
| **Aside from the exposure/intervention, were the groups treated the same?** | Yes  Partial  No  Not reported | Not reported |
| **Detection bias** | | |
| **Were measurements (for exposures or outcomes) carried out and calculated in**  **a standard, valid and reliable way?** | Yes  Partial  No  Not reported | Yes  Assays for testosterone were performed using Diagnostic Products (Los Angeles, CA) Coat-A-Count kits; the interassay coefficients of variation (CVs) were 8% and 5%, respectively. Unbound testosterone was measured by a modification of the procedure of Tremblay and Dube; the interassay CV was 7%. |
| **Were outcome assessors blind to case and control status?** | Yes  Partial  No  Not reported | Not reported |
| **Were all outcomes measured in a standard, valid and reliable way?** | Yes  Partial  No  Not reported | Yes  All subjects were evaluated for 1 night in the sleep laboratory in sound-attenuated, light- and temperature-controlled rooms. During this evaluation, they were continuously monitored for 8 h using 16-channel polygraphs (model 78d, Grass Instrument, Quincy, MA). The three-channel electroencephalogram, three-channel  electrooculogram, and an electromyogram were recorded. The sleep records were subsequently scored independently, according to standardized criteria. Respiration was monitored throughout the night by use of thermocouples at the nose and mouth (model TCT 1R, Grass Instrument) and thoracic strain gauges. All-night recordings of  hemoglobin oxygen saturation (SaO2) were obtained with an oximeter (Model 8800, Noonin Medical, Plymouth, MN) attached to the finger. |
| **Were outcomes assessed objectively and**  **independently?** | Yes  Partial  No  Not reported | Yes  An apnea was considered present if a breath cessation exceeded 10 sec. Each apnea was categorized in terms of obstructive (chest wall movement present) or central (chest wall movement absent). In addition, hypopneas were considered present when a reduction in airflow of approximately 50% was indicated at the nose or mouth and was associated with a reduction of 4% SaO2. OSA was diagnosed using Sleep Disorders Clinic criteria, which employed sleep laboratory plus clinical findings. This diagnosis was made by a Sleep Disorders Medicine specialist (A. N. Vgontzas) based on  whether immediate treatment was considered appropriate. This diagnosis required an apnea/hypopnea index ≥ 10 per hour of sleep plus the presence of clinical symptomatology, e.g. daytime sleepiness, hypertension, or other cardiovascular complication. Daytime sleepiness was assessed subjectively using a sleep  questionnaire on a 4-point scale (none, mild, moderate, or severe). |
| **Attrition bias** | | |
| **What percentage of the individuals recruited into each arm of the study were lost to follow up?** | Yes  Partial  No  Not reported | Not relevant to cross‐sectional study. |
| **What percentage of the individuals were not included in the analysis?** | Yes  Partial  No  Not reported | 54.8% of the women with PCOS. The sleep laboratory sample consisted of 1,000 women. For analysis purposes, compensatory weights were developed to obtain estimates of prevalence of the original target population of women. 452 control patients were included in the study. |
| **Report bias** | | |
| **Is the paper free of selective outcome reporting?** | Yes  Partial  No  Not reported | Yes |
| **Confounding** | | |
| **Are the cohorts comparable on the basis of design or analysis?** | Yes  Partial  No  Not reported | Yes  Controls (n = 452) and PCOS (n = 53) patients were similar in terms of age [32.1 ± 0.3 vs. 30.4 ± 0.9 years, respectively, not significant (NS)], whereas PCOS women were heavier than the controls, as indicated by mean BMI values (38.7 ± 1.1 vs.  26.4 ± 0.3, P < 0.01). |
| **Other bias** | | |
| **Were there any conflicts of interest in the writing or funding of this study?** | Yes  Partial  No  Not reported | Not reported |
| **Was the study sufficiently powered to detect any differences between the groups?** | Yes  Partial  No  Not reported | Not reported |
| **If statistical analysis was undertaken, was this appropriate?** | Yes  Partial  No  Not reported | Yes  For comparisons between two groups, a Student’s t test was used. Odds ratios (ORs) were calculated to evaluate differences between prevalences. To assess which variables were significant predictors of the presence of sleep disordered breathing (SDB) in PCOS women, we used logistic regression analysis, with age, BMI,  testosterone, insulin, and glucose-to-insulin ratio as independent variables. The values are expressed as the mean ± se. All five independent variables were included as continuous variables in this analysis. The statistical confidence level selected for all analyses was P < 0.05. |
| **Comments** | Control subjects were not specifically screened for the presence of PCOS.  PCOS women with sleep problems may be more likely to volunteer to participate.  Control participants were more likely to have been selected if they were at high risk of SDB. | |
| **What is the overall risk of bias?** | Low  Moderate  High Insufficient  information | High |
| **Did risk of bias differ by outcome (eg.**  **primary outcome was low risk but rest**  **were high)?** | No | |

| **Study ID** | Yang et al. 2009 | |
| --- | --- | --- |
| **Study Citation** | Yang HP, Kang JH, Su HY, Tzeng CR, Liu WM, Huang SY. Apnea-hypopnea index in  nonobese women with polycystic ovary syndrome. Int J Gynaecol Obstet. 2009  Jun;105(3):226-9. doi: 10.1016/j.ijgo.2009.02.004 | |
| **Study country** | Taiwan | |
| **Characteristics and external validity – is this study and its results generalizable to my systematic review question?** | | |
| **Patient/population/ participants** | Women with PCOS aged 18–45 years and with a BMI of less than 27 were consecutively recruited after initial screening for PCOS when they presented with oligomenorrhea at the Obstetric and Gynecology Clinic of Taipei Medical University Hospital between May 2006 and January 2007. | |
| **Control population** | Ten age-matched and BMI-matched women who did not have PCOS were recruited as a control group from the same community during the same period. Women were excluded from the control group if they had irregular menstruation or oligomenorrhea, abnormal serum thyroid stimulating hormone or prolactin, or biochemical hyperandrogenemia. | |
| **PCOS diagnostic criteria** | The Rotterdam criteria were used for the initial diagnosis of PCOS. To make the phenotype more consistent, we included patients who had both biochemical hyperandrogenemia and polycystic ovaries. | |
| **N per group** | Women with PCOS (n=18)  Control women (n=10) | |
| **Setting** | Hospital | |
| **Outcomes (primary and other) with definition/tool (eg. self-reported, fasting etc.)** | Primary outcomes:  ‐ AHI≥5 (Polysomnography)  Outcomes not relevant:  ‐ Sleep efficiency, % [Mean (SD)]  ‐ Sleep latency, s [Mean (SD)]  ‐ REM percentage [Mean (SD)]  ‐ REM latency, min [Mean (SD)]  ‐ AHI (total) [Mean (SD)]  ‐ AHI (REM) [Mean (SD)]  ‐ AHI (NREM) [Mean (SD)]  ‐ ARI (total) [Mean (SD)]  ‐ ARI (REM) [Mean (SD)]  ‐ ARI (NREM) [Mean (SD)]  ‐ ARI (spontaneous) [Mean (SD)]  ‐ ARI (PLM-related) [Mean (SD)]  ‐ PLM index, per h [Mean (SD)]  ‐ ESS [Mean (SD)] | |
| **Does the study have a clearly focused question and/or PICO?** | Yes  Partial  No  Not reported | Yes |
| **Inclusion criteria** | Yes  Partial  No  Not reported | Yes  Women aged 18–45 years and with a BMI of less than 27. |
| **Exclusion criteria** | Yes  Partial  No  Not reported | Yes  Exclusion criteria were women who had taken any medication affecting the hypothalamic–pituitary–ovarian axis within the last 6 months, women who had been pregnant within the last year, and women with diabetes, hypertension, other diseases associated with obesity, hyperprolactinemia, abnormal thyroid function tests, and  congenital adrenal hyperplasia. |
| **If there were specified inclusion/exclusion criteria, were these appropriate?** | Yes  Partial  No  Not reported | Partial  Women with comorbidities were excluded which reduces the generalizability of the population. |
| **Is a cross sectional or case-control study the appropriate design to answer this question?** | Yes  Partial  No  Not reported | Yes  This is a cross-sectional study. |
| **Was there sufficient duration of follow-up for outcomes to occur?** | Yes  Partial  No  Not reported | Not relevant (the study is cross-sectional in nature). |
| **Was matching performed?** | Yes  Partial  No  Not reported | Yes  Age and BMI. |
| Summary Result/s | There was no difference between the PCOS and the control groups in any of the other polysomnographic variables. None of the 28 women had an AHI greater than 5, which is the standard for OSA. | |
| **Internal validity – has this study been conducted rigorously in order to reduce bias?** | | |
| **Selection bias** | | |
| **Were the cases and controls taken from comparable populations?** | Yes  Partial  No  Not reported | Partial  Control patients were recruited from the community. However, exposed patients were recruited from the hospital when the presented with oligomenorrhea. |
| **Was the case definition adequate and established in a standard, valid, and**  **reliable way?** | Yes  Partial  No  Not reported | Yes  Total testosterone (TT) was measured by radioimmunoassay using a DSL-4000 kit (Diagnostic System Laboratories, Webster, TX, USA) with a lower limit of sensitivity at 0.08 ng/mL. The inter-assay coefficient of variation (CV) ranged from 8.4% to 9.1%, whereas the intra-assay CV ranged from 7.8% to 9.6%. Androstenedione  (AS) was measured by radioimmunoassay using a DSL-3800 kit (Diagnostic System  Laboratories) with a sensitivity of 0.03 ng/mL. The inter-assay CV ranged from  6.0% to 9.8%, whereas the intra-assay CV ranged from 2.8% to 5.6%. Biochemical hyperandrogenemia was defined as a high serum concentration of TT (N0.8 ng/mL) or AS (N2.44 ng/mL). The presence of polycystic ovaries was determined by pelvic ultrasound performed by a single qualified technician. Hirsutism was confirmed by the same doctor when the Ferriman-Gallwey score was greater than 8. Ferriman Gallwey method scores the degree of hirsutism and reflects the clinical manifestations of hyperandrogenism in patients with PCOS [1,2]. Serum levels of follicle-stimulating hormone (FSH) and luteinizing hormone (LH) were measured by electrochemoluminescence assay (ECLIA). Prolactin (ECLIA, Elecsys 2010 analyzer; Roche Diagnostics, Indianapolis, USA), thyroid stimulating hormone (MEIA technology; Abbott Laboratories, Abbott Park, IL, USA), dehydroepiandrosterone sulfate (radioimmunoassay; Diagnostic System Laboratories), and 17-hydroxyprogesterone (radioimmunoassay; Diagnostic System Laboratories) levels were also evaluated. |
| **Was the control status established in a standard, valid and reliable way?** | Yes  Partial  No  Not reported | Yes  Ten age-matched and BMI-matched women who did not have PCOS were recruited as a control group from the same community during the same period. Women were excluded from the control group if they had irregular menstruation or oligomenorrhea, abnormal serum thyroid stimulating hormone or prolactin, or biochemical  hyperandrogenemia. |
| **Performance bias** | | |
| **Aside from the exposure/intervention, were the groups treated the same?** | Yes  Partial  No  Not reported | Not reported  Insufficient information. |
| **Detection bias** | | |
| **Were measurements (for exposures or outcomes) carried out and calculated in**  **a standard, valid and reliable way?** | Yes  Partial  No  Not reported | Yes  Blood samples were obtained between 08:00 and 10:00 AM after an overnight fast on the third to fifth days of the menstrual cycle or after a progestogen-induced bleed. |
| **Were outcome assessors blind to case and control status?** | Yes  Partial  No  Not reported | Not reported |
| **Were all outcomes measured in a standard, valid and reliable way?** | Yes  Partial  No  Not reported | Yes  All patients were recorded for one full night by standard polysomnography using a computerized sleep-scoring system (Sandman; Tyco Ltd, Ottawa, ON, Canada) in the sleep laboratory of Taipei Medical University Hospital. We recorded 4 channels of the  electroencephalogram (C3/A2, C4/A1, O1/ A2, and Fpz/A1–A2), right and left channels of the electrooculogram, 1 channel of the electrocardiogram (modified V2  lead), 1 channel of submentalis and 2 channels of anterior tibilalis muscles,  and 1 set of chest/abdomen movements. Heart rate and pulse oximetry were  also continuously monitored by a finger probe. Airflow was detected through a nasal cannula pressure transducer and a mouth thermistor. In addition, body position sensors and neck microphones were applied. Whole-night data were analyzed manually in 30-second epochs by the same trained physician and technician. At least 7 hours of  recordings were available for each woman. |
| **Were outcomes assessed objectively and**  **independently?** | Yes  Partial  No  Not reported | Yes  Whole-night data were analyzed manually in 30-second epochs by the same trained physician and technician. At least 7 hours of recordings were available for each woman.  AHI is defined by the number of obstructive apneic and/or hypopneic events lasting more than 10 seconds of sleep that result in either arousal or 4% oxyhemoglobin desaturation. Apnea is defined as a cessation in airflow, whereas hypopnea is defined as a reduction in airflow of 30%. An AHI of 5 or more on polysomnography is defined  as OSA. The total arousal index (ARI) is defined as the number of arousals on the electroencephalogram per hour. Daytime sleepiness was assessed subjectively  using a validated Chinese version of the Epworth Sleepiness Scale (ESS) questionnaire provided by Ning-Hung Chen (Chang Gung Memorial Hospital, Taipei, Taiwan). |
| **Attrition bias** | | |
| **What percentage of the individuals recruited into each arm of the study were lost to follow up?** | Yes  Partial  No  Not reported | Not relevant to cross‐sectional study. |
| **What percentage of the individuals were not included in the analysis?** | Yes  Partial  No  Not reported | 33.33% of the women with PCOS. Twenty-seven women were eligible for inclusion originally. Women who were subsequently found to have undiagnosed diabetes (n=1),  hyperprolactinemia that did not show up on the initial test (n=1), pelvic endometriosis (n=2), women who did not demonstrate polycystic ovaries on pelvic ultrasound (n=3), and women who had only hyperandrogenism but not hyperandrogenemia (n=2) were  also excluded. This left 18 PCOS patients eligible for the study. |
| **Report bias** | | |
| **Is the paper free of selective outcome reporting?** | Yes  Partial  No  Not reported | No  The experimental protocol was approved by the Ethics and Research Committee of the Institutional Review Board for Human Investigation of the Taipei Medical University. |
| **Confounding** | | |
| **Are the cohorts comparable on the basis of design or analysis?** | Yes  Partial  No  Not reported | Yes  Nonobese women with PCOS had a higher waist circumference, waist-to-hip ratio, free androgen index, Ferriman Gallwey score, LH/FSH ratio, serum levels of AS and TT, LH, prolactin, and hsCRP compared with the age- and BMI-matched  control group who did not have PCOS. The women in the PCOS group also had lower  sex hormone-binding globulin levels than the women in the control group. |
| **Other bias** | | |
| **Were there any conflicts of interest in the writing or funding of this study?** | Yes  Partial  No  Not reported | No |
| **Was the study sufficiently powered to detect any differences between the groups?** | Yes  Partial  No  Not reported | No  The study had no outcome event recorded in both the group of women with and without PCOS. |
| **If statistical analysis was undertaken, was this appropriate?** | Yes  Partial  No  Not reported | Yes |
| **Comments** | Low sample size - The study had no outcome event recorded in both the group of women with and without PCOS. | |
| **What is the overall risk of bias?** | Low  Moderate  High Insufficient  information | Moderate |
| **Did risk of bias differ by outcome (eg.**  **primary outcome was low risk but rest**  **were high)?** | No | |
